# Actin polymerization in the endosomal pathway, but not on the *Coxiella*-containing vacuole, is essential for pathogen growth

**DOI:** 10.1371/journal.ppat.1007005

**Published:** 2018-04-18

**Authors:** Heather E. Miller, Charles L. Larson, Robert A. Heinzen

**Affiliations:** Coxiella Pathogenesis Section, Laboratory of Bacteriology, Rocky Mountain Laboratories, National Institute of Allergy and Infectious Diseases, National Institutes of Health, Hamilton, Montana, United States of America; Yale University School of Medicine, UNITED STATES

## Abstract

*Coxiella burnetii* is an intracellular bacterium that replicates within an expansive phagolysosome-like vacuole. Fusion between the *Coxiella*-containing vacuole (CCV) and late endosomes/multivesicular bodies requires Rab7, the HOPS tethering complex, and SNARE proteins, with actin also speculated to play a role. Here, we investigated the importance of actin in CCV fusion. Filamentous actin patches formed around the CCV membrane that were preferred sites of vesicular fusion. Accordingly, the mediators of endolysosomal fusion Rab7, VAMP7, and syntaxin 8 were concentrated in CCV actin patches. Generation of actin patches required *C*. *burnetii* type 4B secretion and host retromer function. Patches decorated with VPS29 and VPS35, components of the retromer, FAM21 and WASH, members of the WASH complex that engage the retromer, and Arp3, a component of the Arp2/3 complex that generates branched actin filaments. Depletion by siRNA of VPS35 or VPS29 reduced CCV actin patches and caused Rab7 to uniformly distribute in the CCV membrane. *C*. *burnetii* grew normally in VPS35 or VPS29 depleted cells, as well as *WASH*-knockout mouse embryo fibroblasts, where CCVs are devoid of actin patches. Endosome recycling to the plasma membrane and *trans*-Golgi of glucose transporter 1 (GLUT1) and cationic-independent mannose-6-phosphate receptor (CI-M6PR), respectively, was normal in infected cells. However, siRNA knockdown of retromer resulted in aberrant trafficking of GLUT1, but not CI-M6PR, suggesting canonical retrograde trafficking is unaffected by retromer disruption. Treatment with the specific Arp2/3 inhibitor CK-666 strongly inhibited CCV formation, an effect associated with altered endosomal trafficking of transferrin receptor. Collectively, our results show that CCV actin patches generated by retromer, WASH, and Arp2/3 are dispensable for CCV biogenesis and stability. However, Arp2/3-mediated production of actin filaments required for cargo transport within the endosomal system is required for CCV generation. These findings delineate which of the many actin related events that shape the endosomal compartment are important for CCV formation.

## Author summary

*Coxiella burnetii*, the human Q fever bacterium, replicates in a harsh, lysosome-like compartment termed the *Coxiella*-containing vacuole (CCV). Here, the pathogen directs formation of the CCV through the activities of secreted type 4B (T4B) effector proteins. Questions remain concerning how the host cytoskeleton and receptor recycling pathways contribute to creation and function of the CCV. We found that filamentous actin (F-actin) patches formed around the CCV in a T4B-dependent manner. Patches were preferred sites of endosome-CCV fusion, a behavior that correlated with patch enrichment of endosome fusion proteins, such as VAMP7 and Rab7. Patch formation required colocalized retromer-WASH-Arp2/3 recycling complexes. In cells depleted of retromer or WASH, CCV lacked actin patches and displayed Rab7, also involved in retromer recruitment, uniformly redistributed around the CCV membrane. *C*. *burnetii* grew normally in these cells, indicating retromer-mediated protein recycling and CCV actin patches are dispensable for productive infection. In contrast, global disruption of Arp2/3-generated F-actin severely restricted CCV formation through a process associated with defective endosome trafficking. We propose that retromer sorting and formation of CCV actin patches are inconsequential to pathogen growth. These studies refine our understanding of vesicular trafficking pathways required for CCV biogenesis.

## Introduction

*Coxiella burnetii* is an intracellular Gram-negative bacterium that causes a severe flu-like disease called Q fever. Q fever is a zoonosis, and transmission to humans typically occurs by inhalation of contaminated aerosols generated by infected ruminant livestock [1]. Following deposition in the lungs, *C*. *burnetii* invades and replicates within alveolar macrophages [2].

*C*. *burnetii* grows within an expansive phagolysosome-like compartment, termed the *Coxiella*-containing vacuole (CCV), that is acidic and displays robust hydrolytic activity [3, 4]. Enlargement of the nascent phagosome to accommodate pathogen growth primarily occurs through fusion with late endosomes/multivesicular bodies, although interactions also occur with autophagic and secretory pathways [5–11]. Endocytic maturation of the CCV and fusion with lysosomes requires sequential engagement of the GTPases Rab5 and Rab7, with Rab7 binding the HOPS (homotypic fusion and vacuole protein sorting) tethering complex [5, 12, 13]. This interaction promotes vesicular fusion by *trans*-SNARE (soluble *N*-ethymaleimide-sensitive factor-attachment protein receptor) complexes, consisting of vesicle-associated membrane protein 7 (VAMP7), Vti1b, syntaxin 7, and syntaxin 8 [10, 14]. Endocytic trafficking to the CCV is further facilitated by clathrin-dependent vesicular transport [15, 16]. An siRNA screen revealed a role for the SNARE syntaxin-17 in mediating homotypic fusion of multiple CCV [11]. Secretion by *C*. *burnetii* of proteins with effector functions by a specialized Dot/Icm type 4B secretion system (T4BSS) is essential for CCV biogenesis, with effectors currently identified that modulate clathrin-mediated vesicular trafficking and autophagosome interactions [8, 15–18].

Several intracellular pathogens exploit the host cytoskeleton for generation, maintenance, and/or stability of their respective replication vacuoles [19–24]. Cytoskeletal requirements for CCV formation are poorly defined, but some information exists on the role of filamentous (F-) actin. Colonne and co-workers [25] described an actin ring circumscribing the mature CCV in THP-1 macrophages that labels with vasodilator-stimulated phosphoprotein (VASP), an actin regulatory protein. Depletion of VASP disrupts CCV formation and heterotypic fusion with latex bead-containing phagosomes. Colonne *at al*. [25] propose that CCV-associated F-actin stabilizes the vacuole, and that actin rearrangements enable vacuole expansion. In HeLa cells, Aguilera and co-workers [26] observed CCV association with F-actin, either as an encompassing ring or in patches. Cells treated with latrunculin B, which disrupts F-actin, exhibit aberrantly small CCVs that fully mature through the endocytic pathway but cannot fuse with latex bead-containing phagosomes. CCVs decorate with the actin-associated small GTPases RhoA and Cdc42. Cells ectopically expressing a dominant-negative form of RhoA display a small, multi-vacuole phenotype. Aguilera *et al*. [26] propose that CCV F-actin participates in membrane transport events.

Actin polymerization regulates late and early stages of endocytic and autophagic trafficking, respectively [27–29]. Important actin regulatory proteins directing polymerization reside in the Wiskott-Aldrich Syndrome protein (WASP) family [30]. These actin nucleation promoting factors (NPFs) activate the actin related protein-2/3 (Arp2/3) complex to nucleate production of barbed actin filaments [30]. The WASP family member Wiskott-Aldrich Syndrome protein and SCAR homolog (WASH) also regulates endosome fission required for recycling of receptors from endosomes to the Golgi apparatus or plasma membrane. Mammalian receptor cargo is sequestered by a WASH-interacting heterotrimeric cargo-selective complex, or retromer, comprised of VPS26A or VPS26B, VPS29, and VPS35 [29, 31–33].

Given that the actin cytoskeleton participates in vesicular trafficking associated with CCV biogenesis, we sought to define the origin and function of CCV F-actin patches. Actin patch formation was a *C*. *burnetii*-driven process. Patches decorated with several proteins involved in fusion of late endosomes, and accordingly, these were preferred sites of CCV fusion. Actin patches also labeled with retromer and WASH complex components, as well as Arp3. Interestingly, inhibition of retromer function reduced actin patches without affecting CCV generation and *C*. *burnetii* growth. Additionally, *C*. *burnetii* grew normally in *WASH*-knockout mouse embryo fibroblasts (MEFs), where CCVs are devoid of actin patches. In contrast, inhibition of the Arp2/3 complex strongly inhibited CCV formation via a process involving endosomal trafficking. These data show that CCV actin patches are not required for vacuole fusion with endocytic vesicles and maintenance of the compartment. However, Arp2/3-driven actin nucleation events that regulate transport through the endosomal system are essential.

## Results

### CCV actin patches are enriched for late endocytic vesicles and fusion regulatory proteins

**Fig 1:**
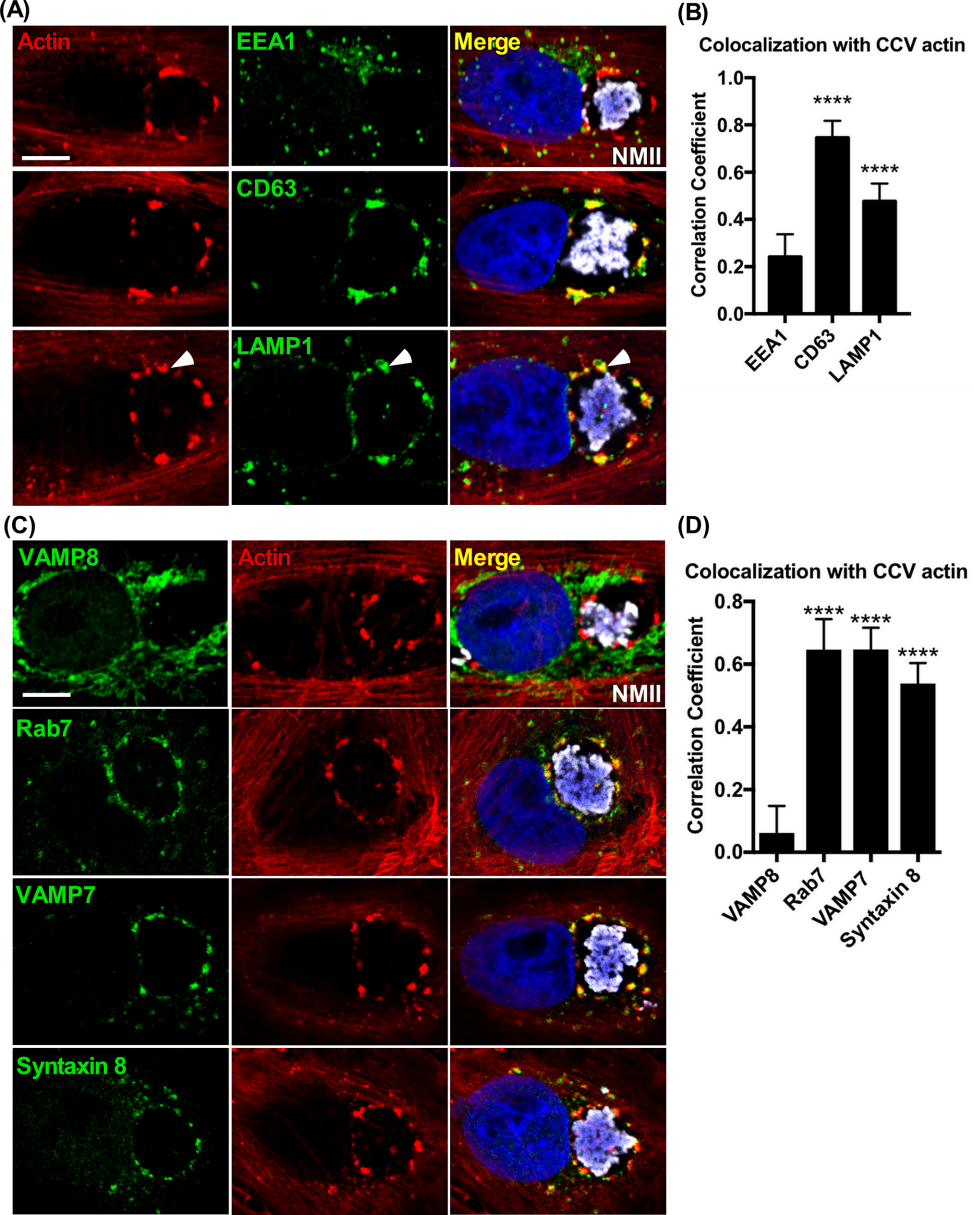
Actin patches on the CCV membrane are enriched for late endocytic vesicles and fusion regulatory proteins. **(A and B)** Colocalization of endosomal components with CCV actin patches. Vero cells fixed at 3 dpi and stained for F-actin, early endosomes (EEA1^+^), late endosomes (CD63^+^), or lysosomes (LAMP1^+^). CCV actin patches cluster with late endocytic markers. Clustering with large, individual vesicles is also seen (white arrows). **(C and D)** Colocalization of fusion regulatory proteins with actin patches. Histograms depict the means ± SD of ≥ 60 cells for at least 3 independent experiments. Statistical significance was determined using Student’s t-test (****P <0.0001). Colocalization was determined using Pearson’s correlation coefficient. NMII, *C*. *burnetii* Nine Mile phase II strain. Scale bar, 5 μm.

Investigating the role of actin in CCV biogenesis revealed patches of F-actin juxtaposed with the CCV membrane **(Fig 1A, S1A Fig)**. CCV actin patches have been previously described [26], but their association with the CCV and function during infection is unclear. Formation and enlargement of the CCV occurs primarily through fusion with late endocytic vesicles [34]. To investigate the role of actin patches in CCV biogenesis, Vero and THP-1 cells were fixed at 3 days post-infection (dpi), a time of accelerated vacuole growth, and stained for F-actin and markers of late endosomes (CD63^+^) and lysosomes (LAMP1^+^). CCV actin patches were associated with CD63^+^ and LAMP1^+^ vesicles that frequently appeared as clusters. Large, individual vesicles were also observed with patches **(Fig 1A and 1B, S1A Fig)**. In contrast, minimal colocalization was observed between CCV actin patches and EEA1, a marker of early endosomes **(Fig 1A and 1B)**.

The interaction between CCV actin patches and late endocytic vesicles suggested that actin patches function in endosomal fusion. Therefore, localization between CCV actin patches and late endosome fusion proteins was assessed. Rab7, a GTPase involved in regulating late endocytic membrane trafficking and fusion, localizes on the CCV membrane and is required for CCV formation and pathogen growth [11, 35]. Staining for Rab7 in Vero cells at 3 dpi revealed Rab7 puncta on the CCV localized with F-actin patches **(Fig 1C and 1D)**. The late endocytic SNAREs, VAMP7 and its partner syntaxin 8, also associate with the CCV membrane, with the former essential for CCV growth [36, 37]. Similar to Rab7, both VAMP7 and syntaxin 8 formed clusters that localized with CCV actin patches, in contrast to VAMP8, a v-SNARE previously shown to be absent on developed CCVs [36] **(Fig 1C and 1D)**. Comparable results were seen in THP-1 cells **(S1B Fig).** Thus, actin patches of expanding CCV accumulate proteins predicted to promote fusion between the CCV and late endosomes. To examine actin patches of more mature CCVs, Vero cells were stained for F-actin and CD63 or VAMP7 at 5 dpi, a time when *C*. *burnetii* is entering stationary phase growth and the CCV has finished expanding [38]. CCV actin patches colocalized with CD63 and VAMP7, but compared to 3 dpi, CCVs had smaller actin patches with decreased CD63 and VAMP7 intensity **(S2A–S2E Fig)**.

### Late endocytic vesicles cluster and fuse with CCV actin patches

**Fig 2:**
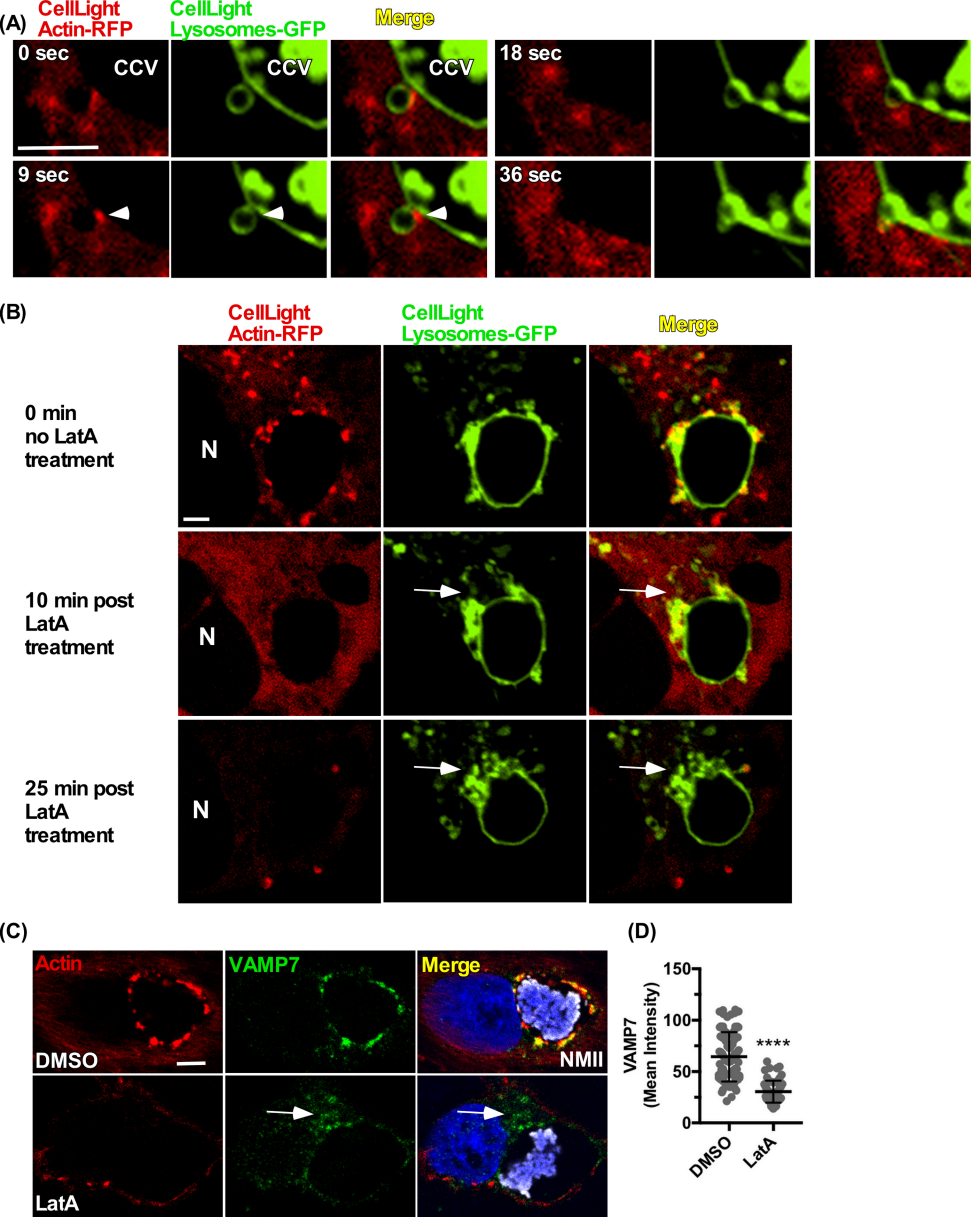
CCV actin patches facilitate vesicle fusion via docking and clustering of late endocytic vesicles to the CCV membrane. **(A)** LAMP1^+^ vesicles fuse with the CCV at actin patches. Live cell images of a Vero cell at 3 dpi expressing lysosome-GFP (LAMP1) and actin-RFP. **(B)** Latrunculin A (LatA) treatment redistributes LAMP1^+^ clusters at actin patches around the CCV to the juxta-nuclear region (white arrows). Live cell images of a Vero cell at 3 dpi expressing lysosome-GFP (LAMP1) and actin-RFP. **(C and D)** LatA treatment redistributes VAMP7^+^ clusters at actin patches to the juxta-nuclear region (white arrows). Vero cells at 3 dpi were treated with LatA for 10 min prior to fixation and immunostaining for F-actin, VAMP7, and NMII. Histogram depicts the mean intensity ± SD of ≥ 60 cells for at least 3 independent experiments. Statistical significance was determined using Student’s t-test (****P <0.0001). NMII, *C*. *burnetii* Nine Mile phase II strain. Scale bar, 2.5 μm.

The role of actin in membrane fusion is not defined; however, actin polymerization is associated with tethering late endocytic vesicles to target membranes [39]. Insight into CCV actin patch function can be gained by live cell imaging. Fusion between endocytic vesicles and CCV actin patches was examined by spinning disk confocal fluorescence microscopy. CellLight reagents were used to express fluorescent proteins that label lysosomes (LAMP1-GFP) and actin (actin-RFP). In Vero cells at 3 dpi, live cell imaging revealed docking of LAMP^+^ vesicles with CCV actin immediately before fusion **(Fig 2A, S1 Movie)**. To investigate whether CCV actin patches are needed for concentration of late endocytic vesicles, 3 dpi Vero cells were treated with latrunculin A (LatA) to depolymerize F-actin. Prior to LatA treatment, clusters of LAMP1^+^ vesicles were associated with F-actin patches punctuating the CCV. After LatA treatment, vesicles translocated to the juxta-nuclear region of the cell, leaving a large portion of the CCV devoid of LAMP1^+^ vesicles **(Fig 2B, S2 Movie)**. CCV CD63 was also redistributed although EEA1-labeled early endosomes were not **(S3A and S3B Fig).** These results suggest actin patches serve as a scaffold that supports fusion of late endocytic vesicles with the CCV. To resolve whether actin polymerization is also needed for SNARE puncta, Vero cells at 3 dpi were treated with LatA, then stained for VAMP7. Consistent with LatA-mediated detachment of late endocytic vesicles, VAMP7 was depleted on the CCV membrane **(Fig 2C and 2D)**. These data indicate actin polymerization promotes late endocytic SNARE clustering on the CCV membrane.

### CCV actin patches depend on type IV secretion of *C*. *burnetii* effectors

**Fig 3:**
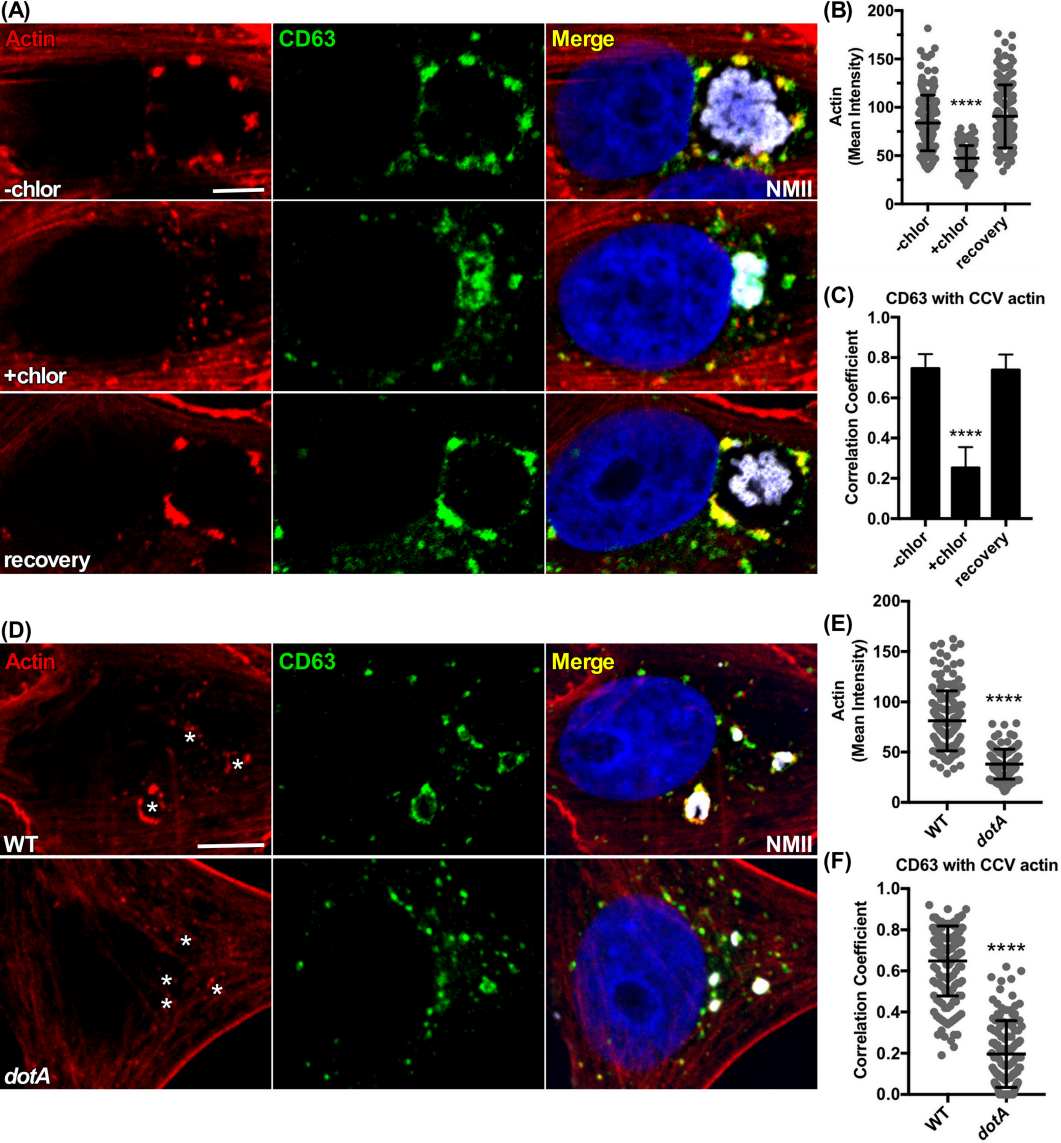
Formation of CCV actin patches depend on secretion of effectors by the *C*. *burnetii* Dot/Icm type 4B secretion system. **(A, B, and C)** Chloramphenicol reversibly causes CCV shrinkage and elimination of actin patches and associated clusters of CD63^+^ vesicles. Vero cells at 2 dpi were treated with chloramphenicol for 24 hr (+chlor) then fixed along with the corresponding 3 dpi untreated control (-chlor). Treated cells at 3 dpi were also washed to remove chloramphenicol and allowed an additional 24 hr recovery before fixation (recovery). Cells were fluorescently stained for F-actin and CD63. **(D, E, and F)** CCV actin patches and colocalization with CD63 are lost in cells infected with a *C*. *burnetii dotA* mutant. Vero cells infected for 24 hr with wild type *C*. *burnetii* or a *dotA* mutant were fixed and stained for F-actin and CD63. Asterisks mark the *C*. *burnetii* vacuole in the actin panels. Histograms depict the mean intensity of CCV actin or area ± SD of ≥ 60 cells for at least 3 independent experiments. Statistical significance was determined using Student’s t-test (****P <0.0001). Colocalization was determined using Pearson’s correlation coefficient. NMII, *C*. *burnetii* Nine Mile phase II strain. Scale bar, 5 μm.

The *C*. *burnetti* Dot/Icm type IVB secretion system (T4BSS) translocates effectors into the host cytoplasm that direct biogenesis of the CCV [17]. To test whether actin patch generation requires *C*. *burnetii* protein synthesis, Vero cells at 2 dpi were treated with chloramphenicol for 24 hr, then stained. Compared to untreated cells at 3 dpi, treated cells exhibited collapsed CCVs that lacked actin patches. Clustering of CD63^+^ vesicles on the CCV was also lost compared to untreated 3 dpi controls. The effects were reversible following washout of chloramphenicol and a 24 hr recovery period **(Fig 3A–3C)**. Similarly, chloramphenicol treatment reversibly decreased CCV clustering and colocalization of VAMP7 and Rab7 with actin **(S4A–S4D Fig)**. To confirm that CCV actin patch formation is T4BSS-dependent, Vero cells were infected with a *C*. *burnetii dotA* mutant and stained for F-actin and CD63 **(Fig 3D–3F)** [17]. At 1 dpi, the *dotA* mutant failed to induce formation of CCV F-actin patches associated with CD63^+^ vesicles. These results demonstrate that polymerization of CCV actin patches requires *C*. *burnetii* protein synthesis and Dot/Icm T4BSS effectors.

### Retromer-associated actin regulators colocalize with CCV actin patches

Actin functions in various endosomal processes, such as endosome biogenesis, maturation, and transport [29, 40–42]. Additionally, actin facilitates fusion between lysosomes and phagosomes [43, 44]. Actin regulatory proteins control production of vesicular F-actin structures. Actin nucleators, such as annexin A2 and Arp2/3, initiate actin filamentation, with Arp2/3 generating branched actin filaments. Arp2/3 is recruited and activated by NPFs, such as N-WASP and WASH. Moesin, an ezrin-radixin-moesin (ERM) family protein, and cortactin, bind and stabilize F-actin structures [40, 41]. Ezrin, an ERM family protein, recruits N-WASP to endosomal membranes to initiate actin polymerization associated with phagosome-lysosome fusion [43]. Conversely, retromer recruits the WASH complex (WASH, FAM21, stumpellin, SWIP, CCD53) via FAM21 to endosomal membranes where it initiates actin polymerization that drives tubule scission and recycling of internalized membrane receptors from endosomes to the *trans*-Golgi (retrograde trafficking) or plasma membrane [31, 33, 42].

**Fig 4:**
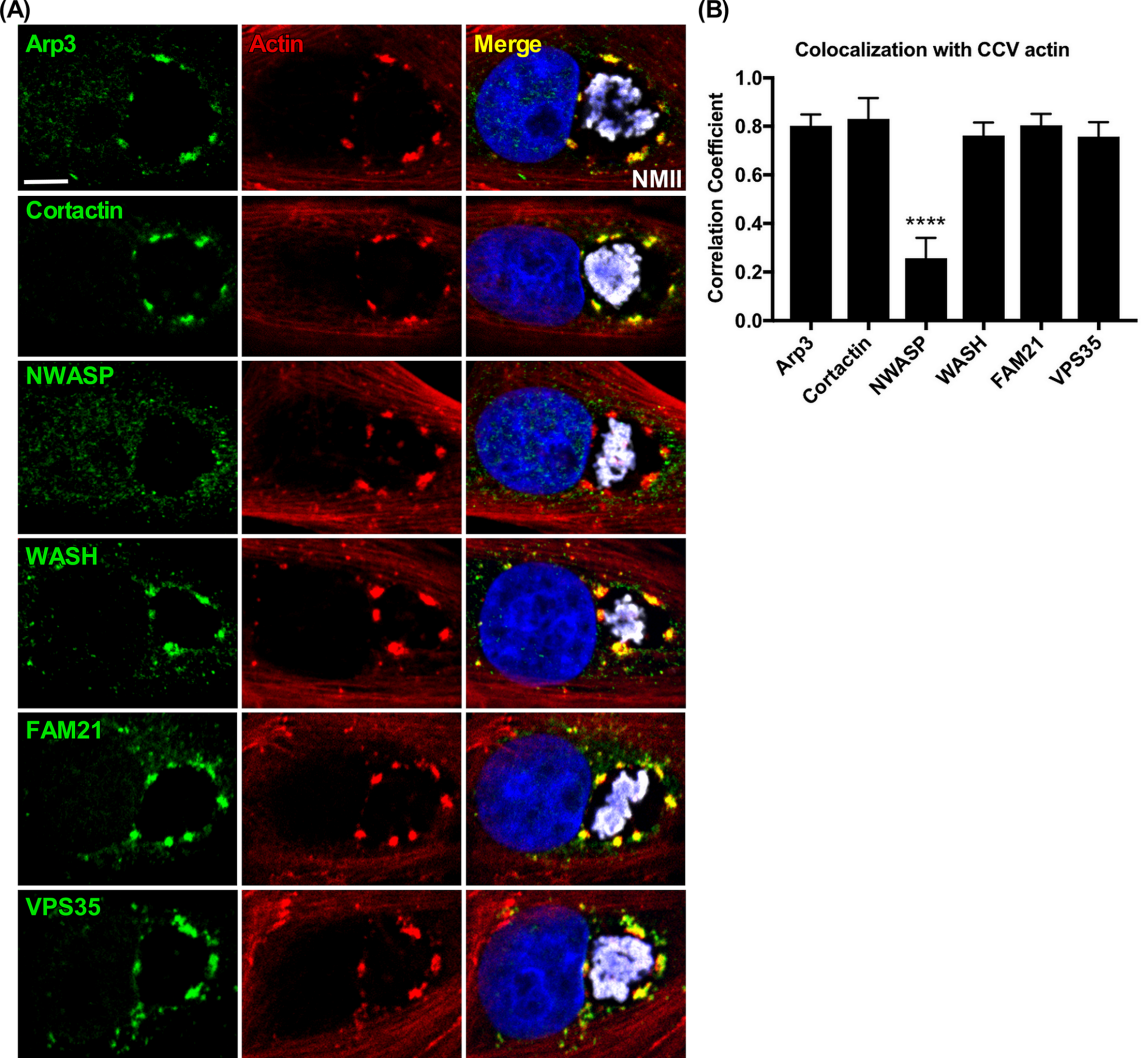
Retromer-associated actin regulators colocalize with CCV actin patches. **(A and B)** CCV actin patches colocalize with Arp3, cortactin, WASH, and FAM21. The retromer protein VPS35 also colocalizes with patches whereas N-WASP does not. Vero cells at 3 dpi were immunostained for the indicated proteins. Histogram depicts the means ± SD of ≥ 60 cells for at least 3 independent experiments. Statistical significance was determined using Student’s t-test (****P <0.0001). Colocalization was determined using Pearson’s correlation coefficient. NMII, *C*. *burnetii* Nine Mile phase II strain. Scale bar, 5 μm.

To identify regulators of actin polymerization at the CCV, Vero and THP-1 cells at 3 dpi were stained for vesicle-related actin regulators. VPS35, FAM21, WASH, Arp3, and cortactin clustered with CCV actin patches, suggesting CCV actin polymerization is regulated by the retromer-WASH complex, with filaments stabilized by cortactin **(Fig 4A and 4B, S5 Fig)**. Arp2 phosphorylation, which is required for activation, was equivalent in infected and uninfected cells [45] **(S6 Fig)**. N-WASP exhibited minimal colocalization with CCV actin patches **(Fig 4A and 4B, S5 Fig)**. Ezrin, moesin, and annexin A2 did not localize to the CCV **(S7A and S7B Fig)**.

To test whether the retromer-WASH complex association with CCV actin was directed by *C*. *burnetii*, Vero cells 2 dpi were treated with chloramphenicol for 24 hr, then fixed and stained for WASH or VPS35 **(S8A and S8B Fig)**. Corresponding to the loss of CCV actin patches following treatment, WASH and VPS35 clusters were also lost, an effect that was reversed following antibiotic washout. Furthermore, CCVs harboring a *dotA* mutant failed to recruit Arp3 **(S9 Fig).** These data suggest *C*. *burnetii* effectors are required for recruitment and clustering of the retromer-WASH complex to mediate polymerization of CCV actin patches.

### Retromer association with late endosomal/lysosomal markers is enriched in infected cells

**Fig 5:**
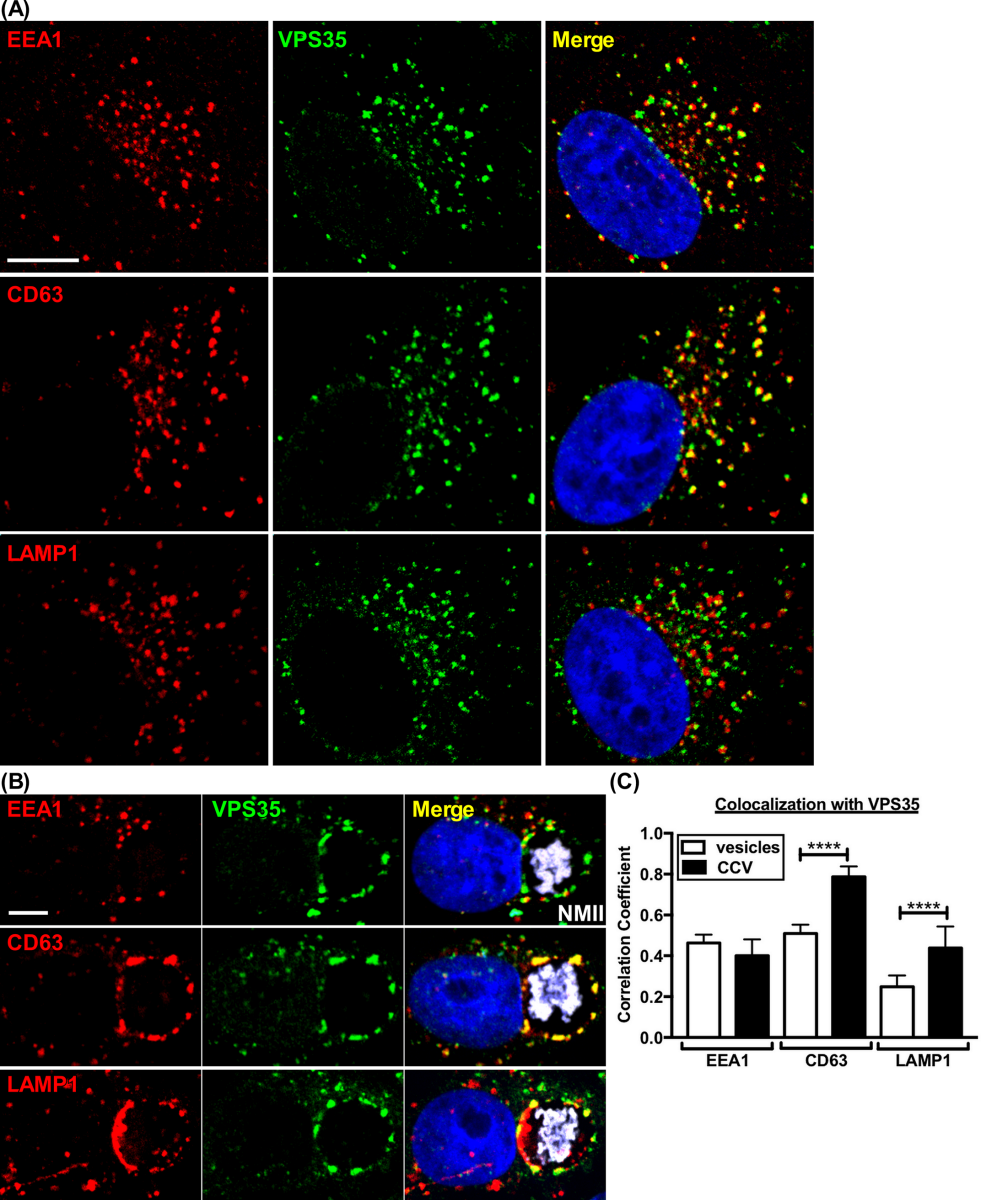
Retromer colocalizes with CD63^+^ vesicles that cluster with CCV actin patches. **(A and C)** Vero cells were immunostained for retromer (VPS35^+^), early endosomes (EEA1^+^), or late endosomes/lysosomes (CD63^+^, LAMP1^+^). EEA1^+^ and CD63^+^ vesicles colocalize similarly with VPS35, while LAMP1^+^ vesicles are much less colocalized. **(B and C)** Vero cells 3 dpi, stained as in (A). Clustered CD63^+^ vesicles on the CCV membrane have high colocalization with VPS35 compared to EEA1^+^ vesicles. LAMP1^+^ vesicles on the CCV membrane also show increased colocalization with VPS35. Colocalization analysis of vesicles and CCVs was determined using Pearson’s correlation coefficient. Graphs represent the means ± SD of ≥ 60 cells from at least 3 independent experiments. Statistical significance determined by Student’s t-test (****P <0.0001). NMII, *C*. *burnetii* Nine Mile phase II strain. Scale bar, 5 μm.

Retromer sequesters membrane receptors for their retrieval from endosomes to the *trans*-Golgi or plasma membrane, a function that typically involves membrane tabulation and fission [31]. In infected cells, retromer also localizes with CCV actin patches associated with endosome fusion. To determine how retromer interacts with vesicles of the endosomal pathway during infection, Vero cells, left uninfected or infected for 3 days, were stained for retromer (VPS35^+^) and early endosomes (EEA1^+^), late endosomes (CD63^+^), or lysosomes (LAMP1^+^). Consistent with previous reports [46, 47], VPS35 in uninfected cells associated with early and late endosomes, but minimally with lysosomes **(Fig 5A and 5C)**. In infected cells, colocalization of VPS35 with markers of late endosomes and lysosomes was increased **(Fig 5B and 5C)**, with an enrichment of label associated with the CCV. Thus, *C*. *burnetii* infection alters the cellular distribution of retromer.

### Sorting nexins partially colocalize with actin patches

Sorting nexins (SNXs) are phosphatidylinositol 3-monophosphate (PI3P) binding proteins that coordinate with retromer to recycle receptors from endosomes. SNX1 or SNX2 dimerize with SNX5 or SNX6 and regulate canonical retrograde trafficking. SNX3 mediates an alternative route of cargo trafficking to the *trans*-Golgi in a WASH and actin-independent manner. SNX27 is involved in recycling plasma membrane receptors. [31, 48]. In contrast to the near complete localization of VPS35 with CCV actin patches, SNXs moderately colocalized **(S10A and S10B Fig)**. To validate the specificity of antibodies directed against SNX1 and SNX2, Vero cells infected for 1 day with *Chlamydia trachomatis* (L2 serotype) were immunostained. As previously reported [49], SNX1 and SNX2 decorated the chlamydial inclusion **(S10C Fig)**.

### Retrograde recycling of the cation-independent mannose-6-phosphate receptor (CI-M6PR) is normal in infected cells

**Fig 6:**
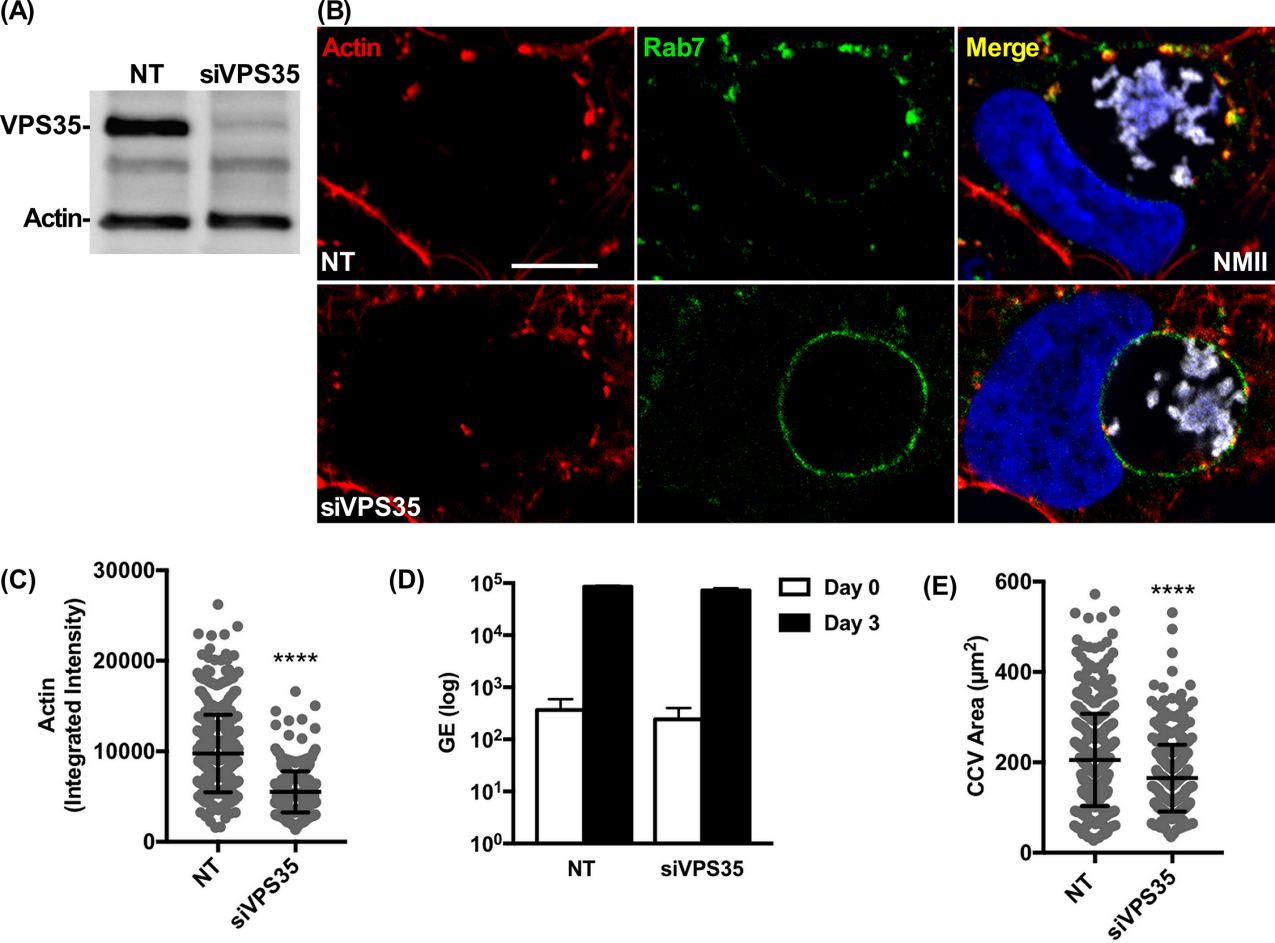
Knockdown of VPS35 reduces CCV actin patches and redistributes Rab7 with no effect on *C*. *burnetii* growth. **(A)** Immunoblot of HEK 293 cells with knockdown of VPS35 by siRNA. A non-targeting pool (NT) siRNA was included. Actin was used as a loading control. **(B, C, D, and E)** Growth of *C*. *burnetii* in siVPS35 or NT-treated HEK 293 cells using qPCR to determine genome equivalents (GE). Three dpi VPS35 knockdown or NT-treated HEK 293 cells fluorescently stained for F-actin and Rab7. Histograms depict the mean intensity of CCV actin or area ± SD of ≥ 60 cells for at least 3 independent experiments. Statistical significance was determined by the Student’s t-test (****P <0.0001). NMII, *C*. *burnetii* Nine Mile phase II strain. Scale bar, 5 μm.

*C*. *trachomatis* and *Legionella pneumophila* inhibit retrograde trafficking via sequestration of SNX5/SNX6 and VPS29, respectively, to the pathogen vacuole [50, 51]. Because retromer and SNXs localized to the CCV membrane, we examined whether *C*. *burnetii* infection disrupts normal retrograde trafficking of CI-M6PR from endosomes to the *trans*-Golgi network. Inhibition of retrograde trafficking traps CI-M6PR in dispersed, early endosomes as opposed to a focused, juxta-nuclear localization within the *trans-*Golgi [47, 52]. CI-M6PR dispersal did not occur upon knockdown (KD) by siRNA of VPS35 [53, 54] **(Fig 6A, S11 Fig).** Therefore, the chemical inhibitor Retro-2 was used which prevents vesicle fusion with the *trans*-Golgi network [55]. Without treatment, uninfected and infected Vero cells after 3 days of incubation showed focused CI-M6PR staining **(S12A and S12B Fig)**. In Retro-2 treated cells, CI-M6PR showed dispersed staining. Collectively, these data indicate infection does not disrupt retrograde recycling of CI-M6PR.

### Knockdown of VPS35 reduces CCV actin patches and redistributes Rab7 with no effect on *C*. *burnetii* growth

Retromer recruits the WASH complex to endosomal membranes where it promotes actin polymerization required for scission of membrane tubules containing cargo for recycling [31, 33, 42]. To investigate whether retromer is necessary for CCV actin patch and vacuole formation, KD of VPS35 was performed **(Fig 6A)**. VPS35 KD inhibited CCV actin patch formation as witnessed by decreased actin patch size and intensity **(Fig 6B and 6C)**. Similar results were seen with KD of VPS29 in HEK 293 cells **(S13A–S13D Fig)**. VPS35 KD cells lacked FAM21 and WASH on the CCV membrane **(S14A–S14D Fig)**, indicating retromer is required for recruitment of the WASH complex to the CCV membrane and for efficient polymerization of CCV actin. Compared to cells treated with non-targeting (NT) siRNA, VPS35KD had no effect on *C*. *burnetii* replication but slightly decreased the size of the 3 day CCV **(Fig 6D and 6E)**.

In addition to regulating fusion of late endosomes, Rab7 recruits retromer by binding VPS35 [56, 57]. Focalized clusters of CD63 and Rab7 on the CCV in control cells were absent in VPS35 KD cells, where the markers localized uniformly around the CCV **(Fig 6B, S15A Fig)**. Diminished actin patches of VPS35 KD cells exhibited reduced colocalization with CD63, suggesting CD63^+^ vesicles are not docking to CCVs at actin patches **(S15A and S15B Fig)**. Similarly, VAMP7 exhibited decreased clustering and colocalization with CCV actin patches compared to NT cells **(S15C and S15D Fig)**. Collectively, these results suggest retromer depletion allows Rab7 to uniformly distribute on the CCV, which promotes a similar distribution of late endocytic vesicles/fusion regulators.

KD of VPS35 perturbed recycling of the glucose transporter 1 (GLUT1) from endosomes to the plasma membrane, with trafficking redirected to lysosomes **(S16A Fig)** [58]. *C*. *burnetii* infection did not disrupt GLUT1 recycling to the plasma membrane. However, KD of VPS35 in infected cells dramatically increased the percentage of GLUT1^+^ CCVs **(S16B and S16C Fig)**. This indicates CCV growth is not dependent on retromer recycling of plasma membrane receptors.

### *WASH* deficiency prevents CCV actin patch formation without affecting *C*. *burnetii* or CCV growth

**Fig 7:**
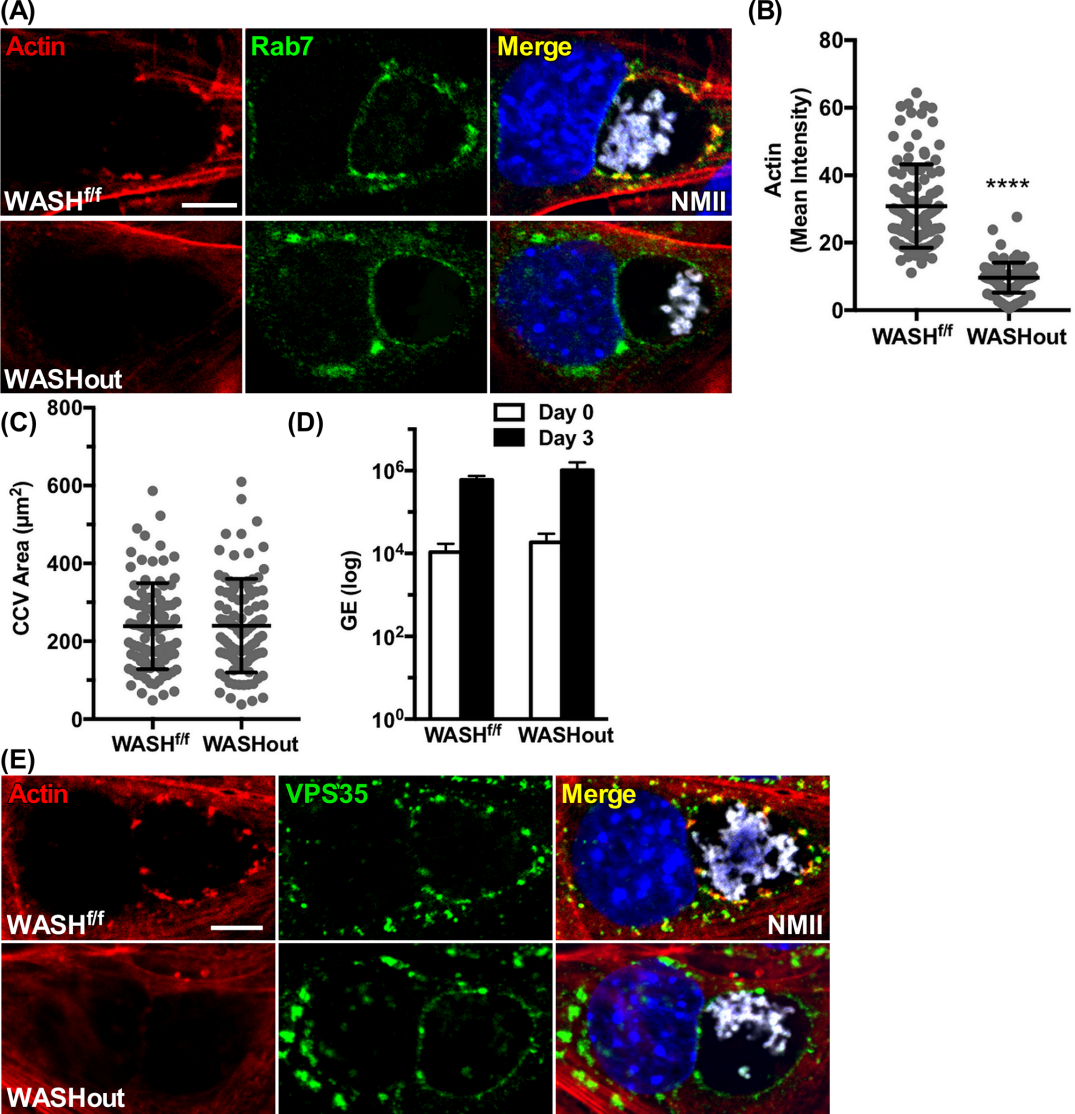
WASH deficiency prevents CCV actin patch formation without affecting *C*. *burnetii* growth or CCV size. **(A, B, and C)** WASHout MEFs (WASH-negative) do not have CCV actin patches and show uniform distribution of Rab7 on the CCV membrane. WASH^f/f^ MEFs untreated or treated with 4-hydroxy-tamoxifen (WASHout) were fixed at 3 dpi and fluorescently stained for F-actin and Rab7. **(D)** Growth of *C*. *burnetii* in WASH^f/f^ or WASHout MEFs using qPCR for determining genome equivalents (GE). Histograms depict the mean intensity of CCV actin or area ± SD of ≥ 60 cells for at least 3 independent experiments. Statistical significance was determined by the Student’s t-test (****P <0.0001). **(E)** WASHout MEFs (WASH-negative) do not have CCV actin patches and show uniform distribution of VPS35. Same treatment regime as (A) but fixed and fluorescently stained for VPS35 and actin. NMII, *C*. *burnetii* Nine Mile phase II strain. Scale bar, 5 μm.

In VPS35 KD cells, the CCV lacks WASH and has reduced actin patches. To determine whether WASH is necessary for CCV actin patch formation and vacuole growth, we performed infections of inducible *WASH* knockout MEFs. Treatment with 4-hydroxy-tamoxifen (4-OHT) of WASH^(f/f)^ MEFs eliminates expression of *WASH* (WASHout) as determined by immunoblot and immunofluorescence **(S17A and S17B Fig)**. Similar to VPS35 KD cells, CCVs in WASHout cells at 3 dpi lacked actin patches and exhibited uniform distribution of Rab7 around the CCV **(Fig 7A and 7B)**. Rab7 redistribution was anticipated as retromer binds Rab7 [47]. CCV size and *C*. *burnetii* replication remained similar to untreated control cells (WASH^f/f^) **(Fig 7C and 7D)**. WASHout cells also displayed uniform staining of VPS35 around the CCV **(Fig 7E)**. Treatment of wild type MEFs (WASH^+/+^) with 4-OHT did not affect actin patch formation, Rab7 clustering, CCV size or *C*. *burnetii* replication **(S18A–S18D Fig)**. Collectively, these results indicate that WASH depletion inhibits CCV actin patch formation, resulting in dispersion of retromer/WASH-generated actin sorting platforms.

### Arp2/3-dependent endocytic trafficking is necessary for CCV biogenesis

**Fig 8:**
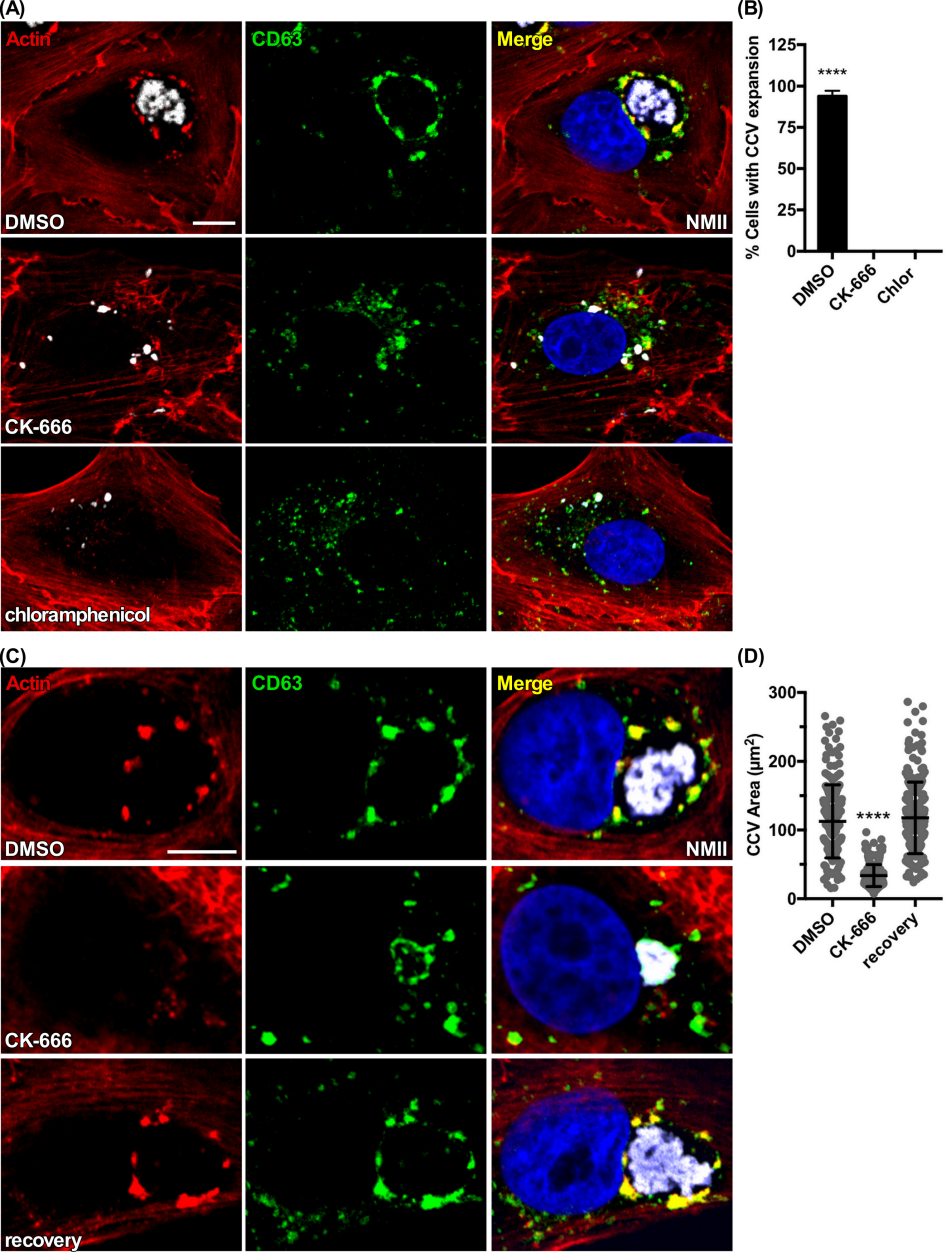
Arp2/3 is needed for CCV biogenesis. **(A and B)** CK-666 treatment prevents CCV expansion. Vero cells at 24 hr post-infection were treated with CK-666 or chloramphenicol for 2 days. Cells were fixed and fluorescently stained for CD63 and F-actin. **(C and D)** CK-666 treatment causes CCV collapse. Vero cells at 2 dpi were treated with CK-666 for 24 hr, then fixed along with the corresponding 3 dpi DMSO-treated control or washed to remove CK-666 and allowed an additional 24 hr recovery before fixation. Cells were fluorescently stained for F-actin and CD63. Histograms depict the means of the percentage of cells with normal CCV expansion or CCV area ± SD of ≥ 60 cells for at least 3 independent experiments. Statistical significance was determined by the Student’s t-test (****P <0.0001). NMII, *C*. *burnetii* Nine Mile phase II strain. Scale bar, 5 μm.

The Arp2/3 complex nucleates formation of barbed actin filaments involved in phagocytic uptake and endosomal trafficking [29]. Expansion and maintenance of the CCV is normal without WASH, an NPF that activates Arp2/3, suggesting nucleation at the CCV is dispensable for *C*. *burnetii* growth [29]. To determine if Arp2/3-mediated actin polymerization in the endosomal pathway is necessary for CCV biogenesis, and to distinguish from phagocytic uptake of *C*. *burnetii*, Vero cells were treated at 1 dpi for 2 days with the specific Arp2/3 inhibitor CK-666, which stabilizes the inactive state of Arp2/3 [59]. Like chloramphenicol-treated cells, CCVs in CK-666-treated cells lacked actin patches and failed to develop into spacious vacuoles supporting *C*. *burnetii* growth **(Fig 8A and 8B)**. Additionally, CCVs in Vero cells infected for 2 days, then treated for 1 day with CK-666, displayed shrunken CCVs, an effect that was reversible **(Fig 8C and 8D)**. Loss of actin patches also correlated with loss of CCV Arp3 labeling **(S19A and S19B Fig)**. KD of Arp 3 also resulted in significantly smaller CCVs **(S19C, S19D and S19E Fig)**. Collectively, these results suggest CCVs of treated cells have a severe reduction in fusion with endosomes. To confirm inhibitory effects were not cell type specific, or related to retromer-WASH regulated trafficking or actin patch formation, WASHout cells at 2 dpi were treated for 1 day with CK-666. Infected WASHout cells also had shrunken CCVs **(S20A and S20B Fig)**. This result indicated Arp2/3-mediated endocytic events upstream of vesicle fusion with CCVs are being disrupted.

**Fig 9:**
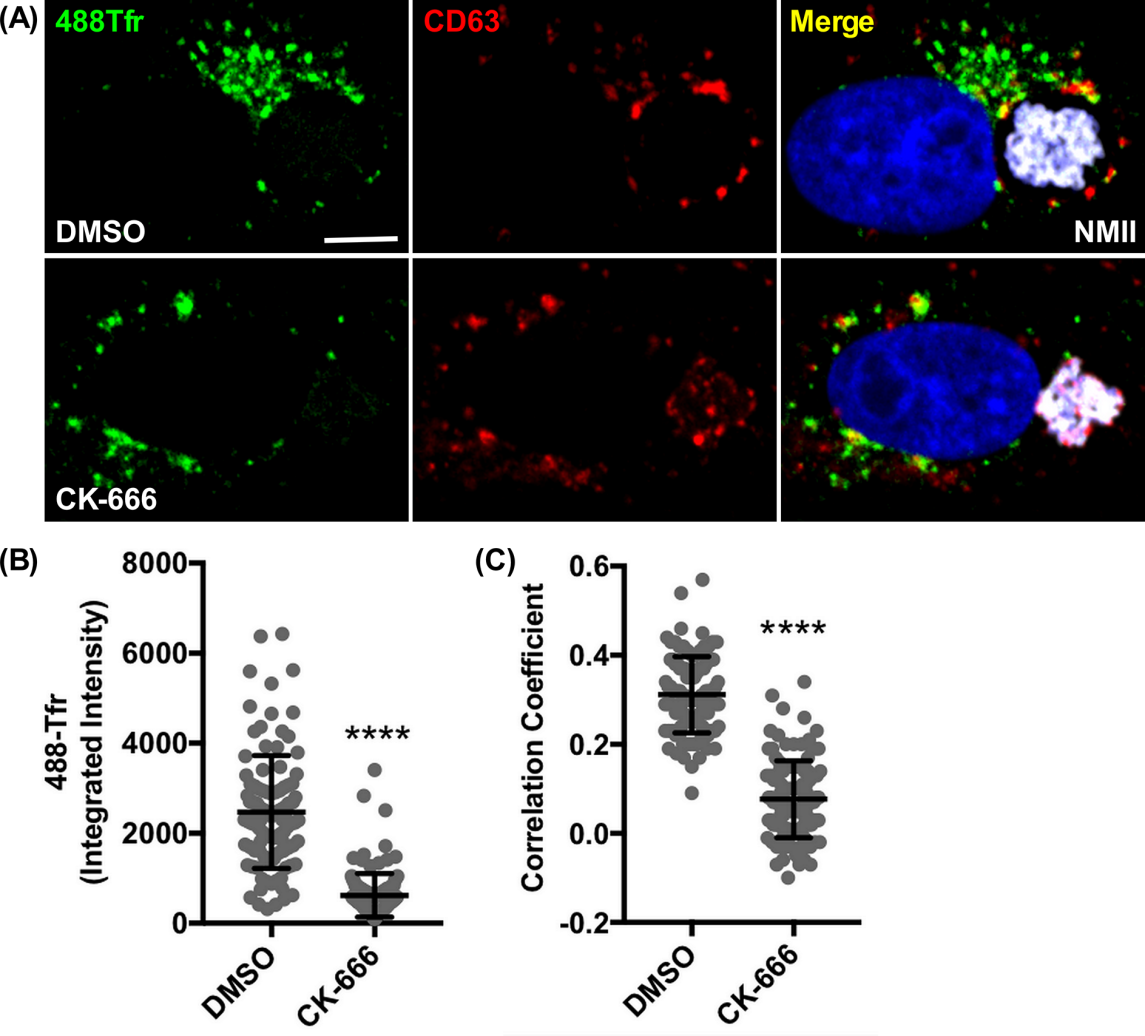
Arp2/3 is required for endocytic trafficking of transferrin in *C*. *burnetii* infected cells. (A-C) Two dpi Vero cells treated with CK-666 or DMSO for 24 hr were incubated with 488-transferrin (488Tfr) for 1 hr, washed with PBS, then fixed and immunostained for CD63 and *C*. *burnetii*. CK-666 inhibits trafficking of 488Tfr to CCVs, decreasing the amount of 488Tfr on the CCV and colocalization with CCV CD63 clusters. Graphs represent the means ± SD of ≥ 60 cells from 3 independent experiments. Statistical significance was determined by the Student’s t-test (****P <0.0001). NMII, *C*. *burnetii* Nine Mile phase II strain. Scale bar, 5 μm.

Fusion with late endosomes is required for CCV biogenesis [36]. Transferrin traffics to the CCV via the endosomal pathway [60]. Therefore, transferrin localization was examined in cells treated with CK-666 to determine if Arp2/3 is required for endosomal trafficking associated with CCV biogenesis. In Vero cells infected for 2 days, then treated with CK-666 for 24 hr, trafficking of 488-transferrin to CD63^+^ limiting membrane of CCVs was severely reduced **(Fig 9A–9C)**. These data demonstrate that Arp2/3-mediated actin dynamics that regulate trafficking within the endosomal pathway are required for CCV formation and pathogen growth.

## Discussion

Exploitation of the actin cytoskeleton is a common theme among intracellular bacteria [61]. Here, we show that F-actin patches form on the CCV membrane in Vero cells and THP-1 macrophages. Patches decorate with late endosome fusion proteins CD63, Rab7, VAMP8, syntaxin 8, and to a lesser extent, LAMP1. Consistent with the presence of membrane fusion complexes, actin patches are preferred docking sites for vesicle fusion. Rapid disruption of CCV actin patches with LatA results in relocalization of LAMP1^+^ vesicles away from the CCV, without affecting vacuole size. Accordingly, this corresponds to the disappearance of CCV VAMP7. Actin patches are less pronounced on non-expanding, mature vacuoles where fusion processes are likely down-regulated.

Our data show that CCV actin patches also colocalize with retromer, Fam21 WASH, and Arp2/3, and that the function of these proteins is necessary for patch formation. Several sorting nexins are also proximal to the retromer, although none strongly colocalize. SNX1 and SNX2 have previously been shown to localize in proximity of WASH and VPS29 [33, 62]. Despite an association with CCV membrane fusion sites, retromer-generated actin is dispensable for CCV formation and *C*. *burnetii* growth. These conclusions are supported by robust replication of *C*. *burnetii* in VPS29 or VPS35 KD cells and *WASH* knockout MEFs, which lack CCV actin patches. Our results contrast with a previous study that reported individual silencing of *VPS29*, *VPS35*, *SNX2*, *SNX3*, *SNX5*, or *SNX6* inhibits *C*. *burnetii* infection. [11]. However, due to functional redundancy of SNX1 and SNX2, as well as SNX5 and SNX6, depletion of one of either pair will not disrupt retrograde trafficking [53, 63]. Retromer is proposed to benefit *C*. *burnetii* by recycling beneficial host factors and/or removing harmful CCV determinants. Promoting proper CCV maturation was also suggested, although acidification of the CCV appears normal [11]. Disparate results may be due, in part, to different experimental readouts and cell lines (e. g. HeLa versus HEK 239 cells).

Unlike the neutral effect of retromer inhibition on *C*. *burnetii* growth, intracellular growth of *Legionella pneumophila*, *C*. *trachomatis*, and *Salmonella typhimurium* is negatively impacted by retrograde trafficking, and each pathogen secretes effector molecules to disrupt the process [49, 51, 64, 65]. *S*. *typhimurium* inhibits retrograde trafficking of CI-M6PR through the activity of the type 3 secretion system (T3SS) effector SifA, which binds the host protein SifA- and kinesin-interacting protein (SKIP). SifA-Skip sequesters Rab9, which is necessary for late endosome to *trans-*Golgi transport of CI-M6PR. The end result is a *Salmonella*-containing vacuole with reduced lysosomal activity that enables pathogen growth [66]. The *C*. *trachomatis* T3SS effector IncE localizes to the chlamydial inclusion where it binds SNX5 and SNX6 [49, 50, 67]. Like *S*. *typhimurium*, subversion of retrograde trafficking is proposed to benefit chlamydial infection by disrupting lysosome function [50, 67]. In *L*. *pneumophila*, the T4BSS effector RidL localizes to the *Legionella*-containing vacuole where it binds VPS29 and PI3P. These interactions restrict assembly of the retromer, thereby inhibiting retrograde trafficking [51]. The benefit to *L*. *pneumophila* is unclear.

SNX1, SNX2, SNX5, and SNX6 contain a Bin/Amphiphysin/Rvs (BAR) domain with curvature sensing, inducing, and stabilizing activities that promote endosomal tubulation associated with recycling of receptors from endosomes to the *trans*-Golgi (retrograde transport) or plasma membrane [48, 63]. These SNXs, as well as SNX3 and SNX27, contain a phox homology (PX) domain that preferentially binds PI3P on early endosomes. The canonical retromer involved in retrograde trafficking of cargo, such as CI-M6PR, is recruited to membranes by binding of VPS35 to both Rab7 and SNX3 [56, 57]. The retromer involved in recycling plasma membrane receptors, such as GLUT1, is recruited via binding of VPS26A or VPS26B to SNX27 [58, 68–70]. Both of these retromer complexes utilize SNX-BAR and WASH-Arp2/3-mediated actin polymerization for tubule formation and scission [31]. The SNX3 retromer traffics cargo such as Wntless to the *trans*-Golgi where it is loaded with Wnt for transport to the plasma membrane [46, 71, 72]. The SNX3 retromer lacks the WASH complex and SNX-BARs, with cargo vesicles budding from the donor membrane through a process involving clathrin [46, 56]. The appearance of GLUT1 on the CCV membrane following VPS35 KD is consistent with trafficking of the receptor to lysosomes for degradation following retromer disruption [58]. Thus, disruption of this recycling pathway, which controls trafficking of additional receptors, such as the monocarboxylate transporter [73], is of little consequence to *C*. *burnetii*. Surprisingly, VPS35 KD did not disperse CI-M6PR to endosomes, suggesting retrograde trafficking is unaltered, and that delivery of hydrolases to the CCV occurs normally.

In HeLa cells, a 6.5 h treatment with Retro-2 inhibits endosome-to-Golgi retrograde trafficking of toxins without affecting trafficking of endogenous cargos, like CI-M6PR [55]. The precise mechanism of inhibition is unknown but is speculated to involve direct inhibition or altered localization of Golgi syntaxin 5. Based on a dispersed endosomal location of CI-M6PR, our results show trafficking of CI-M6PR in Vero cells is inhibited by Retro-2 following a 48 hr treatment. Retro-2 also did not inhibit CCV formation, which further supports the notion that canonical retrograde trafficking is dispensable for *C*. *burnetii* growth. This result also suggests *C*. *burnetii* does not require CI-M6PR-mediated delivery of hydrolases for growth.

F-actin patches generated in a retromer-WASH complex and Arp2/3-dependent fashion are thought to generate force for scission of endosomal tubules [32, 62]. VPS35-bound FAM21 recruits WASH to endosomal membranes [33, 62]. On endosomes, retromer still binds in the absence of WASH, but is redistributed on the endosome membrane. We observed a similar redistribution of VPS35 on the CCV of *WASH* knockout MEFs.

Activation of Arp2/3 by WASP family NPFs and subsequent generation of branched actin filaments modulates several membrane-associated functions including lamellipodia formation, receptor trafficking, endosome tubulation and shape, retrograde trafficking, autophagosome formation, and endocytosis/phagocytosis [30]. Accordingly, KD of Arp3 prior to infection of HeLa cells inhibits phagocytosis of *C*. *burnetii* [11]. Here, we show that treatment with the specific Arp2/3 inhibitor CK-666, or Arp3 KD, inhibits CCV formation. Moreover, CK-666 treatment of cells harboring mature CCV causes vacuole collapse. Because actin filamentation mediates endocytic trafficking [29], it is predicted to contribute to CCV maturation. Indeed, CK-666 inhibition of Arp2/3 inhibits endocytic trafficking of transferrin to the CCV.

A previous study demonstrated *C*. *burnetii* protein synthesis is required for CCV recruitment of VAMP7 [36]. We show that chloramphenicol reversibly inhibits recruitment of VAMP7, Rab7, VSP35, and WASH, as well as actin patch formation. Moreover, vacuoles harboring a *dotA* mutant lack actin patches. Collectively, these data suggest that recruitment of retromer and membrane fusion complexes is mediated by a *C*. *burnetii* T4BSS effector protein(s). The CCV is unusual in being positive for both Rab7, a marker of late endosomes/lysosomes, and enriched for PI3P, a characteristic of early endosomes [9]. Indeed, enrichment of PI3P on the CCV is associated with the activity of the *C*. *burnetii* effector CvpB. CvpB inhibits phosphotidylinositol 3-phosphate 5-kinase, the enzyme responsible for converting PI3P to phosphatidylinositol 3,5-bisphosphate on late endosomes [9]. Treatment with wortmannin, a phosphatidylinositol 3-kinase inhibitor, releases retromer and SNX1 from endosomes [47, 63]. Thus, the possibility exists that retromer is aberrantly targeted to the CCV based on cooperative binding of VPS35 and SNX3 to Rab7 and PI3P, respectively, without serving a functional role [42, 70].

The ability of CCVs devoid of actin patches, such as those in *WASH* knockout MEFs, to expand normally may be based on the dual function of Rab7 in membrane fission and fusion. In yeast, the Rab7 homolog Ypt7 is released upon membrane tubulation mediated by the SNX-BAR retromer, allowing interaction with the HOPS complex and membrane fusion [74]. Perhaps dispersal of Rab7 following depletion of VPS29 or VPS35, or in *WASH* knockout cells, reflects the lack of F-actin organizing platforms that concentrate Rab7 at membrane fusion complexes [70]. Indeed, actin polymerization organizes WASH in discrete membrane domains [75]. Fusion of late endosomes/MVBs with the CCV would then occur in a dispersed manner, as opposed to localized fusion associated with actin patches. The ability of *C*. *burnetii* to prosper without CCV actin may also be related to the hyper-fusogenic nature of the vacuole [76].

During the preparation of this manuscript, two controversial studies were coordinately published indicating SNX-BAR without retromer can mediate retrograde retrieval of CI-M6PR [53, 54]. Data derived by quantitative proteomics and protein binding assays show CI-M6PR directly binds SNX5 and SNX6 [53, 54]. Moreover, retrograde retrieval of CI-M6PR is unaffected by KD or knockout of retromer components [53, 54]. This behavior is associated with SNX-BAR and retromer occupying separate microdomains on endosomes, as suggested by a previous report [77, 78]. As with our investigation, these studies employed the broadly used and accepted assay for dysfunctional retrograde sorting, i. e., dispersal of CI-M6PR into endosomes. Others have suggested that disparate results arise from different measures of CI-M6PR dispersal [79]. Moreover, a two-step model is proposed that invokes a role for retromer in transiently concentrating cargo, which is then rapidly transferred to SNX-BAR for tubule-mediated retrieval to the Golgi [53, 77]. Clearly, more study is needed, although our data showing poor colocalization of SNXs with retromer, and the lack of CI-M6PR dispersal following VSP35 KD, agrees with the newly proposed model. Nonetheless, plasma membrane recycling of GLUT1 is clearly inhibited upon retromer disruption to result in aberrant trafficking of the receptor to the CCV membrane.

Based on the bulk of published data, we propose a model, similar to one recently proposed by Jimenez-Orgaz *et al*. [80], that invokes a dual regulatory role for CCV Rab7 in endosome fusion and retromer recruitment **(S21 Fig)**. Membrane receptors, such as GLUT1, are recycled from transitioning early to late endosomes by retromer. This can occur coincidently with late endosome engagement with the CCV, but prior to vesicle fusion. Retromer recruitment by CCV Rab7, and subsequent WASH-Arp2/3 generation of actin sorting platforms, concentrates Rab7 and impedes its lateral diffusion. Consequently, recruitment of HOPs/SNARE complexes by Rab7 results in focused fusion of late endosomes with the CCV. In the absence of retromer and sorting platforms, membrane receptors are not recycled but instead deposit on the CCV in a dispersed manner due to the unfocused nature of Rab7. While retromer-derived F-actin is dispensable for *C*. *burnetii* growth, additional Arp2/3-generated F-actin structures are essential for CCV biogenesis and pathogen replication, including those that mediate endocytic trafficking. An intriguing question is the identity and function of TBSS effector(s) modulating this fusogenic behavior. In summary, this study illustrates the complex interplay between the CCV and actin-mediated vesicular trafficking pathways. It also describes a large vacuole model for studying retromer function, reminiscent of vacuoles generated by ectopic expression of constitutively-activated Rab5 [54].

## Materials and methods

### Cell culture and infection

HEK 293 (ATCC CRL-1573, human embryonic kidney epithelial) cells were cultured in Delbecco’s Modified Eagle Medium (DMEM) (Life Technologies) supplemented with 10% fetal bovine serum (FBS). THP-1 (ATCC TIB-202, human monocytic) and Vero (ATCC CCL-81, African green monkey epithelial) cells were cultured in RPMI medium 1640 (Life Technologies) supplemented with 10% FBS. WASH conditional knockout mouse embryonic fibroblasts (MEFs) (Daniel Billadeau, Mayo Clinic) were grown in DMEM containing 10% FBS. Generation and characterization of these cells are described in Gomez *et al*. [81]. MEFs were grown in DMEM containing 10% FBS, and knockout of WASH was obtained with two sequential 24 hr treatments of 3 μM 4-OHT (Sigma, H7904). After treatment, MEFs were washed and cultured for an additional 6–7 days before use in experiments [81]. All cell lines were incubated at 37°C with 5% CO_2_. Infection of Vero cells with *C*. *trachomatis* was at a multiplicity of infection (MOI) of 50 based on inclusion forming units.

*C*. *burnetii* Nine Mile phase II, RSA439 (NMII) was propagated in Vero cells and purified as described [82]. The *C*. *burnetii dotA* mutant was propagated in the synthetic medium ACCM-2 as described [17]. *C*. *trachomatis* (LGV-434, serotype L2) was propagated in HeLa cells and purified as described [83]. For infection, HEK 293, Vero, or MEF cells were seeded on coverslips in 24-well plates at a density of 4 x 10^4^ cells per well. THP-1 cells were seeded on coverslips in 24-well plates at a density of 3 x 10^5^ cells per well and stimulated with 200 nM phorbol myristate acetate (Sigma) for 24 hr for differentiation into macrophage-like cells and attachment to coverslips. For immunofluorescence staining, cells were infected at an MOI of 100 based on genome equivalents (GE) quantified by TaqMan qPCR using a StepOnePlus Real-Time PCR system (Applied Biosystems) and primers specific to *C*. *burnetii groEL* [38]. For *C*. *burnetii* growth analysis, cells were infected at an MOI of 10.

### Inhibitors

Latrunculin A (LatA; Sigma) was used at 1 μg/ml, CK-666 (Sigma) at 200 μM, chloramphenicol at 50 μg/ml, and Retro-2 (Sigma) at 40 μM. All treatments were at 37°C with 5% CO_2_ in RPMI media 1640 (Life Technologies) plus 10% FBS. DMSO was used as a control treatment as necessary.

### Live imaging

Vero cells on glass bottoms of 24-well SensoPlates (Greiner bio-one, 662892) were transfected with CellLight Actin-RFP, BacMam 2.0 (Molecular Probes, C10502) 1 dpi and CellLight Lysosomes-GFP, BacMam 2.0 (Molecular Probes, C10507) the night before imaging at 3 dpi. CellLight reagents were used at concentrations recommended by the manufacturer. Cells were washed to remove CellLight reagents before imaging on a Nikon ECLIPSE Ti spinning disk confocal fluorescence microscope. For imaging of LAMP1^+^ vesicles fusing with CCV membranes, cells were incubated at 37°C with 5% CO_2_ and imaged at 1 frame every 2 sec. For LatA (Sigma) treatment, cells were imaged at room temperature at 1 frame per min. Imaging started 15 min before adding LatA followed by 30 min post-treatment.

### Immunofluorescence staining

Cells were fixed with 4% paraformaldehyde in phosphate-buffered saline (PBS) for 30 min at room temperature and simultaneously permeabilized and blocked for 30 min with 0.1% Triton X-100 or 0.05% saponin plus 1% BSA. Antibodies (3–5 μg/ml) were diluted in Triton or saponin buffers and samples stained for 30–60 min. The following antibodies were used for immunofluorescence and/or immunoblotting: Actin (Abcam, ab8224), Annexin A2 (clone: D11G2, cell signaling, 8235), Arp2 (Abcam, AB128934), phosphoArp2 (Abcam, AB119766), Arp3 (Millipore, 07–272), CD63 (clone: H5C6, BD Pharmingen, 556019), Cortactin (clone: 30/Cortactin, BD Transduction Laboratories, 610049), EEA1 (Cell Signaling, 2411), EEA1 (clone: 14/EEA1, BD Transduction Laboratories, 610456), Ezrin (Cell Signaling, 3145), FAM21C (Millipore, ABT79), GLUT1 (Abcam, ab15309), LAMP1 (Abcam, ab24170), LAMP1 (clone: 1D4B, Santa Cruz, sc-19992), Moesin (clone:EP1863Y, Abcam, ab52490), Moesin (clone: 38/Moesin, BD Transduction Laboratories, 610401), Rab7 (clone: D95F2, Cell Signaling, 9367), SNX1 (clone: 51/SNX1, BD Transduction, 611482), SNX2 (clone: 13/SNX2, BD Transduction, 611308), SNX3 (Abcam, ab56078), SNX27 (clone: 1C6, Abcam, ab77799), Syntaxin 8 (clone: 48/syntaxin 8, BD Transduction, 611352), TNG46 (Sigma, T7576), VAMP7 (US Biological, V2024), VAMP8 (Synaptic System, 104302), VPS29 (Sigma, HPA039748), and VPS35 (Abcam, ab10099). Rabbit anti-WASH VCA domain-specific antibodies were kindly provided by Dr. Daniel Billadeau [81]. Rabbit anti-human WASH and guinea pig anti-human N-WASP antibodies were kind gifts from Dr. Matthew Welch [29]. Anti-*C*. *burnetii* antibodies were generated in guinea pigs or rabbits, and anti-*C*. *trachomatis* in rabbits. Alexa Fluor-647, 568, and 488-conjugated secondary antibodies (Life Technologies) were used. For staining F-actin, BODIPY 558/568 or Alexa Fluor-647 labeled phalloidin (Life Technologies) were used. Nuclei were stained with Hoescht 33342 (ThermoFisher). Post-staining, cells were again fixed with 4% paraformaldehyde for 30 min and coverslips mounted using Prolong Gold antifade mountant (ThermoFisher).

### siRNA transfection

Dharmacon ON-TARGETplus SMARTpool siRNAs for VPS29 (L-009764-01-0005), VPS35 (L-010894-00-0005), Arp3 (L-012077-00-0005), and non-targeting (D-001810-10-5) were used. HEK 293 cells were used as opposed to HeLa cells because more complete and consistent KD was obtained. For KDs, HEK 293 cells were grown on coverslips in 24-well plates at a density of 4 x 10^4^ cells per well for 1 day before transfection. Dharmafect 1 (Dharmacon) was used to transfect cells with siRNA complexes at a concentration of 2 μM. For KD of VPS29 and VPS35, cells were infected two days post-transfection. For KD of Arp3, cells were transfected 1 and 3 days before infection. Cells were fixed 3 dpi and processed for immunofluorescence. Immunoblots of lysates were used to confirm efficiency of KD. Actin served as a loading control.

### *C*. *burnetii* growth in MEFs and HEK 293 cells

MEF or HEK 293 cells seeded at 4 x 10^4^ cells per well in 24-well plates were infected with *C*. *burnetii* at an MOI of 10 by centrifuging at 500 x *g* for 30 min at room temperature. Cells were then washed and incubated with growth medium. Samples were collected by trypsinization, bead beaten with 0.1 mm Zirconia/silica beads (BioSpec) in a homogenizer (ThermoElectron FastPrep FP120), and boiled 10 min. GE were quantified by qPCR.

### CI-M6PR retrograde trafficking

CI-M6PR retrograde trafficking assays were performed as described in Osborne *et al*. [52]. HEK 293 cells were incubated with 10 μg/ml anti-CI-M6PR (BioRad, MCA2048) in serum-free medium for 1 hr at 37°C. Cells were washed with PBS prior to immunostaining. Vero cells were treated with DMSO or Retro-2 for 2 days, followed by incubation for 1 hr at 37°C with 10 μg/ml anti-CI-M6PR in serum free medium. Cells were washed with PBS, then fixed and fluorescently stained.

### Transferrin endocytosis assay

Vero cells seeded on glass coverslips at 4 x 10^4^ cells per well in 24-well plates were infected at an MOI of 100. Two dpi cells were treated with DMSO or CK-666 for 24 hr. Cells were incubated with 25 μg/ml of Alexa Fluor-488 transferrin (ThermoFisher, T13342) for 1 hr, washed three times with PBS, fixed with 4% PFA, then fluorescently immunostained for CD63 and *C*. *burnetii*.

### Microscopy and analysis

Fixed and stained cells were imaged using Zeiss LSM-710 confocal fluorescence microscope (Carl Zeiss). For compiling images, z-sections of 0.32 μm slices were taken. Depicted images of cells are maximum intensity projections of 3 slices, totaling 1 μm thickness. Fiji (Image J, National Institutes of Health) was used for all image analysis. The entire CCV was selected for co-localization and fluorescence intensity analysis and included a combined measurement of 3 z-sections from the vertical center (z-axis) of the CCV. The area of CCVs was measured using CD63, LAMP1, or Rab7 as CCV membrane markers. For determining disruption of CI-M6PR retrograde trafficking, the percentage of cells with visually compact versus scattered CI-M6PR staining was quantitated. Correlation coefficients (Pearson’s Correlation Coefficient) were determined using ImageJ Coloc 2. Unless otherwise stated, a minimum of 60 cells for each condition from 3 independent experiments was used for analyses. GraphPad Prism (GraphPad Software) was used for all graphing and statistics.

## Supporting information

S1 Fig Actin patches on the CCV membrane are enriched for late endocytic vesicles and fusion regulatory proteins in THP-1 cells. **(A)** THP-1 cells 3 dpi were fluorescently stained for F-actin, late endosomes (CD63^+^), or lysosomes (LAMP1^+^). White arrows indicate a single LAMP1^+^ vesicle interacting with actin on the CCV membrane (top panel) and large CCV actin patches colocalized with CD63^+^ vesicle clusters (bottom panels). **(B)** Three dpi THP-1 cells were fixed and stained for F-actin and the indicated fusion regulatory proteins. NMII, *C*. *burnetii* Nine Mile phase II strain. Scale bar, 5 μm.
(TIF)

S2 Fig CCV actin patches decrease with maturation of the CCV. **(A-C)** Three and 5 dpi Vero cells were stained for F-actin and CD63, then analyzed for CCV area and actin fluorescence intensity. Actively growing 3 dpi CCVs have larger actin patches and greater F-actin mean intensity compared to larger 5 dpi CCVs. **(D and E)** Same as (A), but stained and analyzed for VAMP7. Larger and more mature CCVs at 5 dpi have decreased staining for VAMP7, suggesting less fusion with late endosomes. Graphs represent the means ± SD of ≥ 60 cells of at least 3 independent experiments. Statistical significance was determined by the Student’s t-test (****P <0.0001). NMII, *C*. *burnetii* Nine Mile phase II strain. Scale bar, 5 μm.
(TIF)

S3 Fig CCV actin patches facilitate vesicle fusion via docking and clustering of late endocytic vesicles to the CCV membrane. **(A and B)** Vero cells 3 dpi were treated for 10 min with LatA, then fixed and stained for actin and CD63 or EEA1. Latrunculin A (LatA) treatment redistributes CD63^+^ clusters at actin patches around the CCV to the juxta-nuclear region. LatA treatment did not redistribute EEA1+ early endosomes. Scale bar, 5 μm.
(TIF)

S4 Fig Chloramphenicol treatment eliminates CCV actin patches/fusion protein platforms. **(A and B)** Two dpi Vero cells treated with 50 μg/ml chloramphenicol for 24 hr (+chlor) were fixed along with the corresponding 3 dpi untreated controls (-chlor), or washed to remove chloramphenicol and allowed an additional 24 hr recovery before fixation. Cells were stained for F-actin and VAMP7. Chloramphenicol treatment eliminates actin patches and colocalization with VAMP7 (middle panel). **(C and D)** Same as (A and B), with Rab7 staining. The colocalization of CCV actin patches with Rab7 is reduced with chloramphenicol treatment. Colocalization analysis of CCVs was determined using Pearson’s correlation coefficient. Graphs represent the means ± SD of ≥ 60 cells from at least 3 independent experiments. Statistical significance determined by Student’s t-test (****P <0.0001). NMII, *C*. *burnetii* Nine Mile phase II strain. Scale bar, 5 μm.
(TIF)

S5 Fig Retromer-associated actin regulators colocalize with CCV actin patches in THP-1 cells. Three dpi THP-1 cells were immunostained for the indicated proteins. Colocalization of CCV actin patches is high for cortactin, Arp3, and the Arp2/3-promoting factors WASH and FAM21. The retromer protein VPS35 also colocalizes with patches. N-WASP shows no colocalization. NMII, *C*. *burnetii* Nine Mile phase II strain. Scale bar, 5 μm.
(TIF)

S6 Fig Arp2 phosphorylation is similar between infected and uninfected cells. Immunoblot of lysates of infected or uninfected Vero cells grown for 3 days. The blot was probed with anti-phosphoArp2 antibody, then striped and probed anti-Arp2 antibody.
(TIF)

S7 Fig Ezrin, moesin, and annexin 2 are absent in CCV actin patches. **(A)** Vero cells 3 dpi were immunostained for ezrin, moesin, or annexin 2. Ezrin and moesin have no colocalization with CCV actin patches whereas annexin 2 has poor colocalization that was not above background staining. **(B)** Same as (A), but with THP-1 cells. Neither ezrin, moesin, nor annexin 2 colocalize with CCV actin patches. NMII, *C*. *burnetii* Nine Mile phase II strain. Scale bar, 5 μm.
(TIF)

S8 Fig *C*. *burnetii* protein synthesis is required for CCV actin polymerization and clustering of WASH and VPS35. **(A)** Two dpi Vero cells treated with 50 μg/ml chloramphenicol for 24 hr were fixed along with the corresponding 3 dpi untreated controls, or washed to remove chloramphenicol and allowed an additional 24 hr recovery before fixation. Cells were stained for WASH and F-actin. Chloramphenicol treatment eliminates actin patches and WASH clusters on the CCV membrane (middle panel). **(B)** Same as (A) but stained for retromer component VPS35. VPS35 clustering on the CCV is also lost with chloramphenicol treatment. NMII, *C*. *burnetii* Nine Mile phase II strain. Scale bar, 5 μm.
(TIF)

S9 Fig CCVs harboring a *dotA* mutant lack Arp3. Vero cells infected for 24 hr with wild type *C*. *burnetii* or a *dotA* mutant were fixed and stained for CD63 and Arp3. Asterisks mark CCVs in Arp3 and CD63 panels. Histogram depicts the mean intensity of CCV Arp3 ± SD of ≥ 60 cells for at least 3 independent experiments. Statistical significance was determined using Student’s t-test (****P <0.0001). NMII, *C*. *burnetii* Nine Mile phase II strain. Scale bar, 5 μm.
(TIF)

S10 Fig Sorting nexins display partial colocalization with retromer and CCV actin patches. **(A and B)** Three dpi Vero cells were fixed, fluorescently stained for F-actin, and immunostained for the indicated sorting nexins (SNXs). SNXs show partial colocalization with CCV actin patches. **(C)** For a positive SNX control, Vero cells 1 dpi with *Chlamydia trachomatis* L2 were stained for SNX1 or SNX2. Similar to a previous report [48], SNX1 and SNX2 stain the *C*. *trachomatis* inclusion membrane, validating the antibodies. Colocalization analysis of CCVs was determined using Pearson’s correlation coefficient. Graphs represent the means ± SD of ≥ 60 cells from at least 3 independent experiments. Statistical significance was determined by the Student’s t-test (****P <0.0001). NMII, *C*. *burnetii* Nine Mile phase II strain. Scale bar, 5 μm.
(TIF)

S11 Fig CI-M6PR is not dispersed in VPS35 knockdown cells. HEK 293 cells knocked down for VPS35 or non-targets and infected for 3 days were incubated with anti-CI-M6PR antibody for 1 hr at 37°C, then fixed and immunostained. The *trans*-Golgi (TNG46) was stained to evaluate retrograde trafficking of CI-M6PR. Knockdown of VPS35 (siVPS35) has no effect on CI-M6PR staining which shows focused colocalization with TGN46 similar to the non-targeted control. Images are representative of 2 independent experiments. NMII, *C*. *burnetii* Nine Mile phase II strain. Scale bar, 10 μm.
(TIF)

S12 Fig *C*. *burnetii* infection does not disrupt retrograde transport of CI-M6PR to the *trans*-Golgi. **(A)** Uninfected or 3 dpi Vero cells treated with DMSO or Retro-2 for 2 days (added 1 dpi) were incubated with anti-CI-M6PR antibody for 1 hr, then fixed and immunostained. **(B)** Images in (A) were analyzed for focused and dispersed CI-M6PR staining. In contrast to highly dispersed staining of CI-M6PR in Retro-2 treated cells, untreated infected and uninfected cells display focused peri-nuclear staining. Graphs represent the means ± SD of ≥ 50 cells from 3 independent experiments. NMII, *C*. *burnetii* Nine Mile phase II strain. Scale bar, 10 μm.
(TIF)

S13 Fig Knockdown of VPS29 repeats knockdown of VPS35. **(A)** Immunoblot of HEK 293 cells transfected with siRNA for VPS29 (siVPS29) or a non-targeting pool (NT). Actin was used as a loading control. **(B-D)** Three dpi VPS29 knockdown or NT treated HEK 293 cells were fluorescently stained for F-actin and Rab7. Knockdown of VPS29 redistributes Rab7 on the CCV and CCV actin patch size and intensity are decreased along with a slight reduction in CCV area. Graphs represent the means ± SD of ≥ 60 cells from 3 independent experiments. Statistical significance was determined by the Student’s t-test (****P <0.0001). NMII, *C*. *burnetii* Nine Mile phase II strain. Scale bar, 5 μm.
(TIF)

S14 Fig Recruitment of WASH complex to the CCV membrane is lost upon knockdown of VPS35. **(A and B)** Three dpi siVPS35 or NT treated HEK 293 cells were fixed and fluorescently stained for F-actin or FAM21. VPS35 knockdown cells have decreased FAM21 intensity on the CCV membrane. **(C and D)** Same as (A and B) but stained for WASH. Knockdown of VPS35 also reduces WASH intensity on the CCV membrane. Graphs represent the means ± SD of ≥ 60 cells from 3 independent experiments. Statistical significance determined by the Student’s t-test (****P <0.0001). NMII, *C*. *burnetii* Nine Mile phase II strain. Scale bar, 5 μm.
(TIF)

S15 Fig Association of CCV actin patches with late endocytic vesicles is reduced in VPS35 knockdown cells. **(A and B)** VPS35 knockdown or control HEK 293 cells at 3 dpi were fluorescently stained for F-actin and CD63. VPS35 knockdown cells have uniform staining of CD63 around the CCV and lack colocalization of CD63 clusters with reduced CCV actin patches. **(C and D)** Same as (A and B) but with VAMP7 staining. VAMP7 staining is still observed around CCVs of VPS35 knockdown cells but lacks colocalization with reduced CCV actin patches. Graphs represent the means ± SD of ≥ 60 cells from 3 independent experiments. Colocalization was determined using Pearson’s correlation coefficient. Statistical significance was determined by the Student’s t-test (****P <0.0001). NMII, *C*. *burnetii* Nine Mile phase II strain. Scale bar, 5 μm.
(TIF)

S16 Fig *C*. *burnetii* infection does not disrupt GLUT1 retromer-dependent trafficking to the plasma membrane. **(A)** Uninfected VPS35 knockdown or NT-treated HEK 293 cells were stained for GLUT1 and CD63. As previously reported [57], knockdown of VPS35 disrupts recycling from early endosomes to the plasma membrane, redirecting GLUT1 trafficking to late endosomes/lysosomes. **(B and C)** Three dpi VPS35 knockdown or NT-treated HEK 293 cells were fixed and stained for GLUT1. Infection alone does not affect GLUT1 trafficking. However, knockdown of VPS35 results in trafficking of GLUT1+ vesicles to the CCV, increasing GLUT1 intensity on the CCV membrane. Graphs represent the means ± SD of ≥ 60 cells from 3 independent experiments. Statistical significance was determined by the Student’s t-test (****P <0.0001). NMII, *C*. *burnetii* Nine Mile phase II strain. Scale bar, 5 μm.
(TIF)

S17 Fig Knockout of WASH in WASH^f/f^ MEFs by 4-hydroxy-tamoxifen treatment. **(A)** Immunoblot of lysates of control wild type MEFs (WASH^+/+^), untreated or treated with 4-hydroxy-tamoxifen (4-OHT), and WASH^f/f^ MEFs, untreated or treated with 4-OHT (WASHout). Actin served as a loading control. **(B)** Three dpi MEFs fluorescently stained for F-actin and WASH. WASHout MEFs do not have CCV actin patches, but still support *C*. *burnetii* growth. NMII, *C*. *burnetii* Nine Mile phase II strain. Scale bar, 5 μm.
(TIF)

S18 Fig 4-hydroxy-tamoxifen treatment has no effect on *C*. *burnetii* infection of WT MEFs. **(A)** Wild type WASH^+/+^ MEFs untreated or treated with 4-OHT were fixed 3 dpi and fluorescently stained for F-actin and Rab7. Treatment of 4-OHT had no effect on CCV actin patch formation or Rab7 clustering on the CCV. **(B and C)** F-actin intensity and areas of CCVs from images of (A). Untreated and 4-OHT treated MEFs have similar CCV actin patch intensity and CCV areas. **(D)** Growth analysis of *C*. *burnetii* in WASH^+/+^ MEFs untreated or treated with 4-OHT using qPCR for determining genome equivalents (GE). No difference in *C*. *burnetii* replication is observed with 4-OHT treated cells. Graphs represent the means ± SD of ≥ 50 cells from 3 independent experiments. NMII, *C*. *burnetii* Nine Mile phase II strain. Scale bar, 5 μm.
(TIF)

S19 Fig Arp2/3 localization is altered by treatment with CK-666. **(A)** Vero cells at 2 dpi were treated with CK-666 for 1 day. Cells were fixed and fluorescently stained for actin and Arp3. **(B)** Histogram depicts the integrated intensity of CCV Arp3 ± SD of ≥ 60 cells for at least 3 independent experiments. Statistical significance was determined using Student’s t-test (****P <0.0001). NMII, *C*. *burnetii* Nine Mile phase II strain. Scale bar, 5 μm. **(C)** Immunoblot of HEK 293 cells with knockdown of Arp3 by siRNA. A non-targeting pool (NT) siRNA was included. Actin was used as a loading control. **(D)** Three dpi Arp3 knockdown or NT-treated HEK 293 cells fluorescently stained for CD63. **(E)** Histogram depicts the CCV area ± SD of ≥ 60 cells for at least 3 independent experiments. Statistical significance was determined by the Student’s t-test (****P <0.0001). NMII, *C*. *burnetii* Nine Mile phase II strain. Scale bar, 5 μm.
(TIF)

S20 Fig Arp2/3 acts upstream of CCV fusion with late endosomes to promote CCV biogenesis. **(A and B)** WASH^+/+^ or WASH^f/f^ MEFs untreated or 4-OHT treated were incubated with DMSO or CK-666 2 dpi for 24 hr, then fluorescently stained for LAMP1 and F-actin. The area of individual CCVs in CK-666 treated cells was measured and compared to DMSO-treated cells. CCVs in WASHout MEFs, which do not require actin patches for lysosomal fusion, have reduced CCV area with CK-666 treatment. Graph represents the means ± SD of ≥ 60 cells from 3 independent experiments. Statistical significance was determined by the Student’s t-test (****P <0.0001). NMII, *C*. *burnetii* Nine Mile phase II strain. Scale bar, 5 μm.
(TIF)

S21 Fig Retromer influences endosome interactions with the CCV and clustering of fusion proteins. The proposed model depicts a dual regulatory role for Rab7 in mediating recruitment of retromer for membrane receptor recycling and fusion of late endosomes with the *Coxiella*-containing vacuole (CCV). In the presence of retromer and WASH (left of dashed line), GLUT1 on transitioning early to late endosomes is normally recycled to the plasma membrane. GLUT1 is recognized by SNX27, which along with Rab7, recruits the retromer-WASH-Arp2/3 complex that generates F-actin required for recycling. Simultaneously, Rab7 recruits the retromer-WASH-Arp2/3 complex which generates F-actin sorting platforms that cluster Rab7 on the CCV. In turn, HOPs/SNARE complexes (VAMP7 and syntaxin 8) are recruited to platforms which results in focused fusion of late endosomes with the CCV. In the absence of retromer and actin sorting platforms (right of dashed line), the unfocused nature of Rab7 results in GLUT1 receptors that are not recycled but instead deposit on the CCV. Late endosomal markers also disperse in the CCV due to the unfocused nature of Rab7.
(TIF)

S1 Movie Actin patches tether late endocytic vesicles to the CCV membrane for fusion. Live cell imaging of a 3 dpi Vero cell transfected with CellLight reagents (BacMam) lysosome-GFP (LAMP1) and actin-RFP. Cells were incubated at 37°C with 5% CO_2_ and imaged at 1 frame every 2 sec.
(AVI)

S2 Movie Dispersal of CCV actin patches releases late endocytic vesicles from the CCV membrane. Live cell imaging of a 3 dpi Vero cell transfected with CellLight reagents (BacMam) lysosome-GFP (LAMP1) and actin-RFP treated with 1 μg/ml of latrunculin A. Cells were imaged at room temperature at 1 frame per min. Imaging started 15 min before adding latrunculin A followed by a 30 min post-treatment.
(AVI)
