# Supplementary figures and images for: Actin polymerization in the endosomal pathway, but not on the Coxiella-containing vacuole, is essential for pathogen growth
Source: PLoS Pathog. 2018 Apr 18;14(4):e1007005. doi: 10.1371/journal.ppat.1007005 (PMC5927470; doi:10.1371/journal.ppat.1007005)

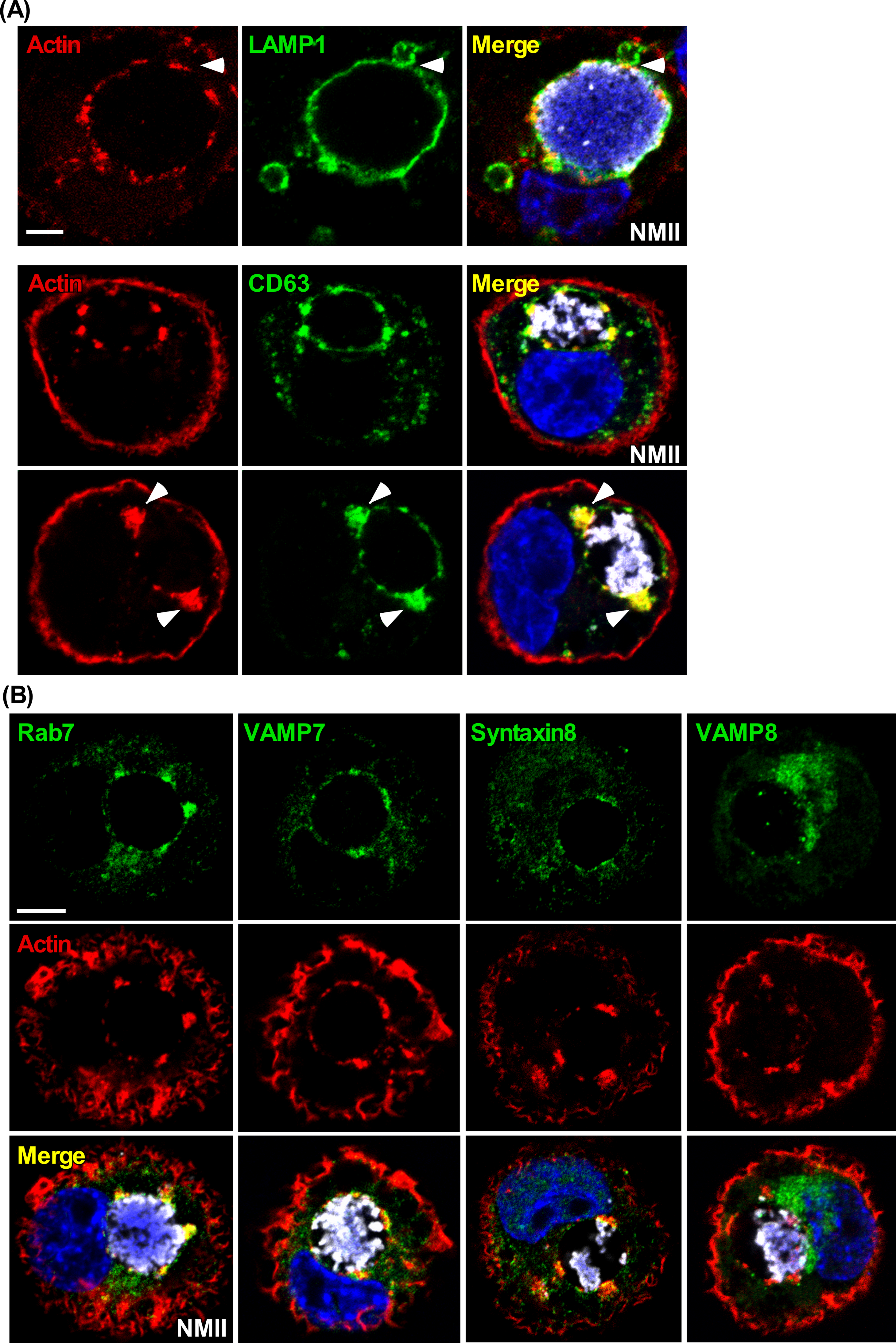

Supplement: S1 Fig — (A) THP-1 cells 3 dpi were fluorescently stained for F-actin, late endosomes (CD63+), or lysosomes (LAMP1+). White arrows indicate a single LAMP1+ vesicle interacting with actin on the CCV membrane (top panel) and large CCV actin patches colocalized with CD63+ vesicle clusters (bottom panels). (B) Three dpi THP-1 cells were fixed and stained for F-actin and the indicated fusion regulatory proteins. NMII, C. burnetii Nine Mile phase II strain. Scale bar, 5 μm. (TIF) [file ppat.1007005.s001.tif]

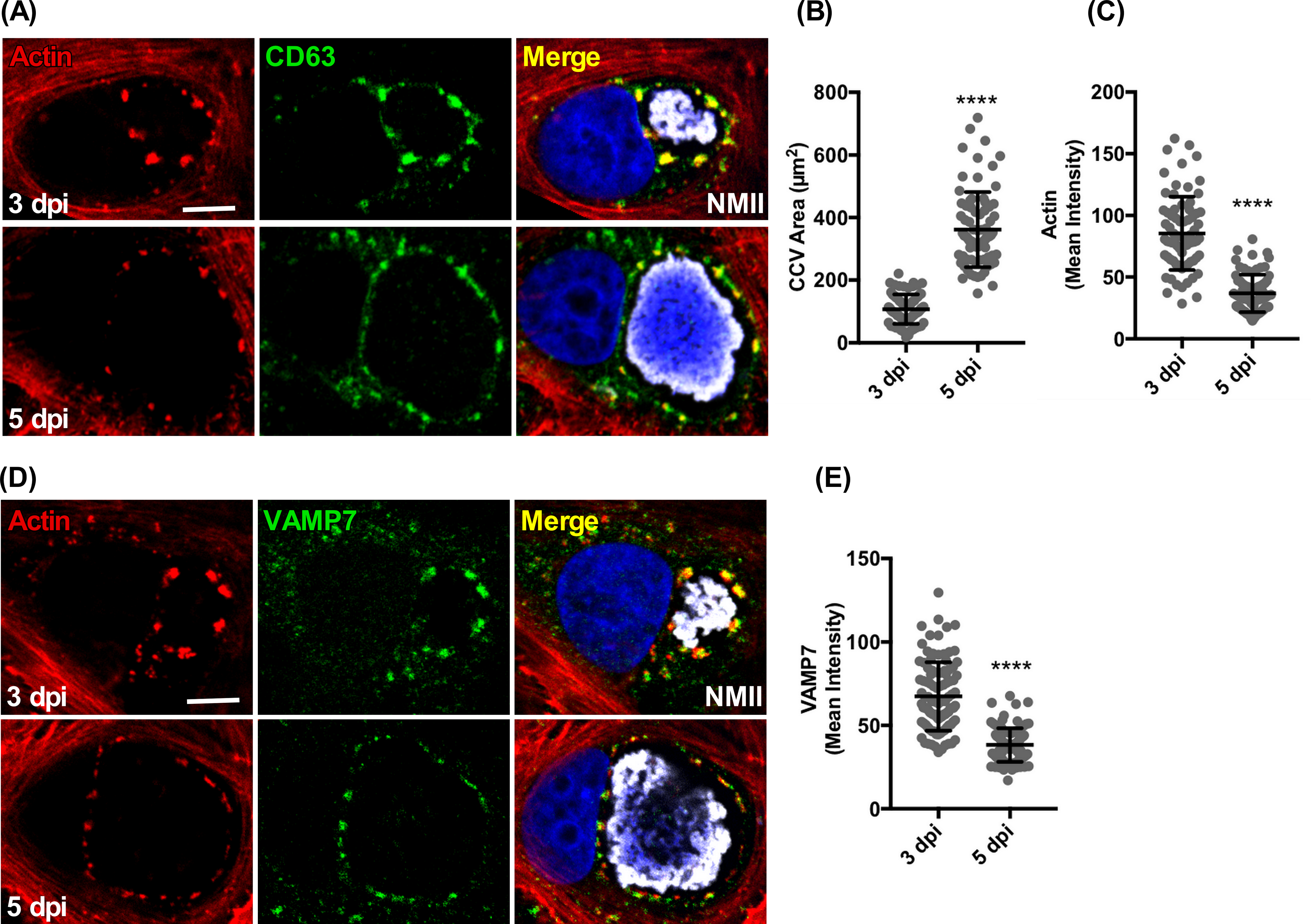

Supplement: S2 Fig — (A-C) Three and 5 dpi Vero cells were stained for F-actin and CD63, then analyzed for CCV area and actin fluorescence intensity. Actively growing 3 dpi CCVs have larger actin patches and greater F-actin mean intensity compared to larger 5 dpi CCVs. (D and E) Same as (A), but stained and analyzed for VAMP7. Larger and more mature CCVs at 5 dpi have decreased staining for VAMP7, suggesting less fusion with late endosomes. Graphs represent the means ± SD of ≥ 60 cells of at least 3 independent experiments. Statistical significance was determined by the Student’s t-test (****P <0.0001). NMII, C. burnetii Nine Mile phase II strain. Scale bar, 5 μm. (TIF) [file ppat.1007005.s002.tif]

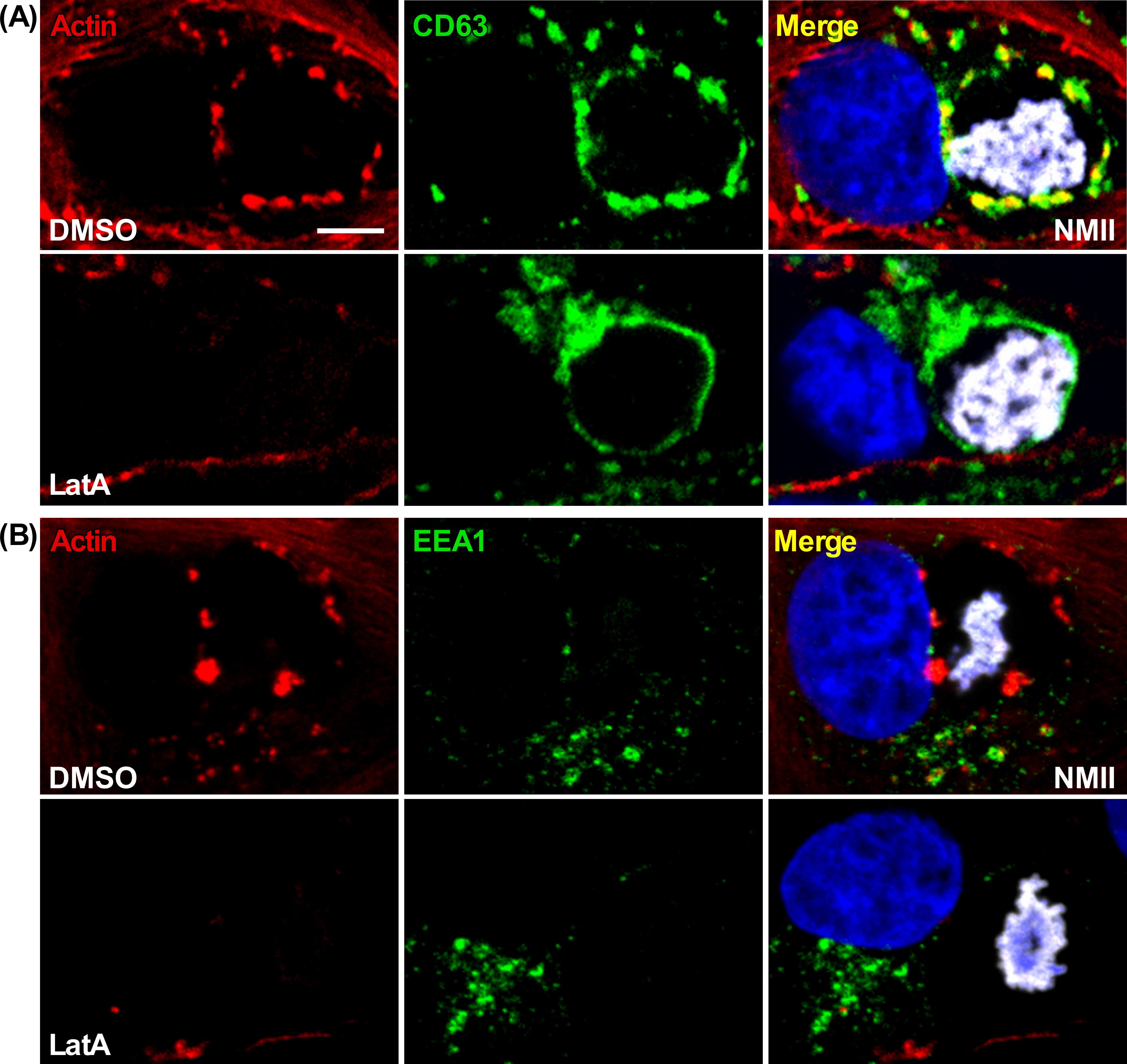

Supplement: S3 Fig — (A and B) Vero cells 3 dpi were treated for 10 min with LatA, then fixed and stained for actin and CD63 or EEA1. Latrunculin A (LatA) treatment redistributes CD63+ clusters at actin patches around the CCV to the juxta-nuclear region. LatA treatment did not redistribute EEA1+ early endosomes. Scale bar, 5 μm. (TIF) [file ppat.1007005.s003.tif]

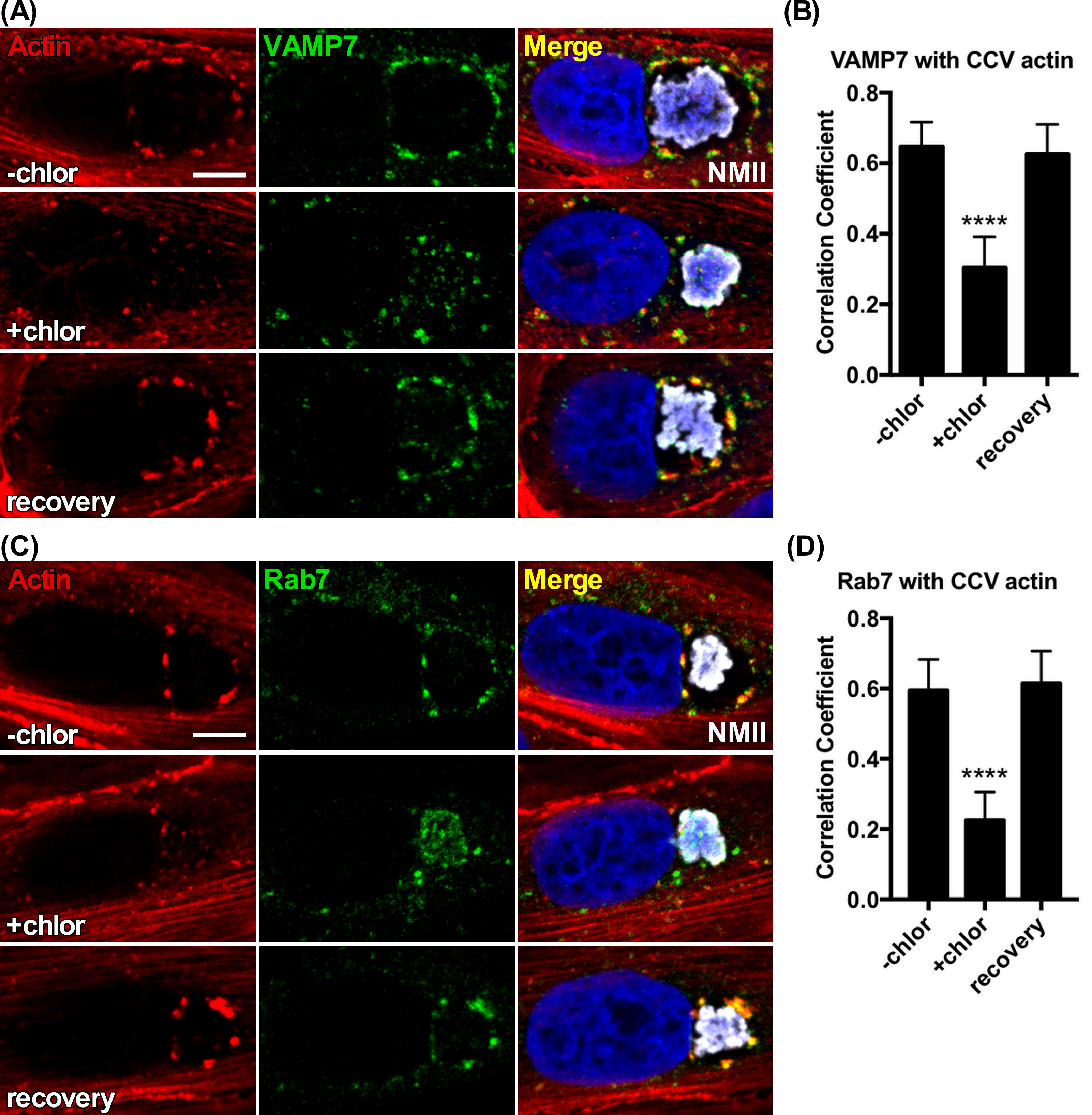

Supplement: S4 Fig — (A and B) Two dpi Vero cells treated with 50 μg/ml chloramphenicol for 24 hr (+chlor) were fixed along with the corresponding 3 dpi untreated controls (-chlor), or washed to remove chloramphenicol and allowed an additional 24 hr recovery before fixation. Cells were stained for F-actin and VAMP7. Chloramphenicol treatment eliminates actin patches and colocalization with VAMP7 (middle panel). (C and D) Same as (A and B), with Rab7 staining. The colocalization of CCV actin patches with Rab7 is reduced with chloramphenicol treatment. Colocalization analysis of CCVs was determined using Pearson’s correlation coefficient. Graphs represent the means ± SD of ≥ 60 cells from at least 3 independent experiments. Statistical significance determined by Student’s t-test (****P <0.0001). NMII, C. burnetii Nine Mile phase II strain. Scale bar, 5 μm. (TIF) [file ppat.1007005.s004.tif]

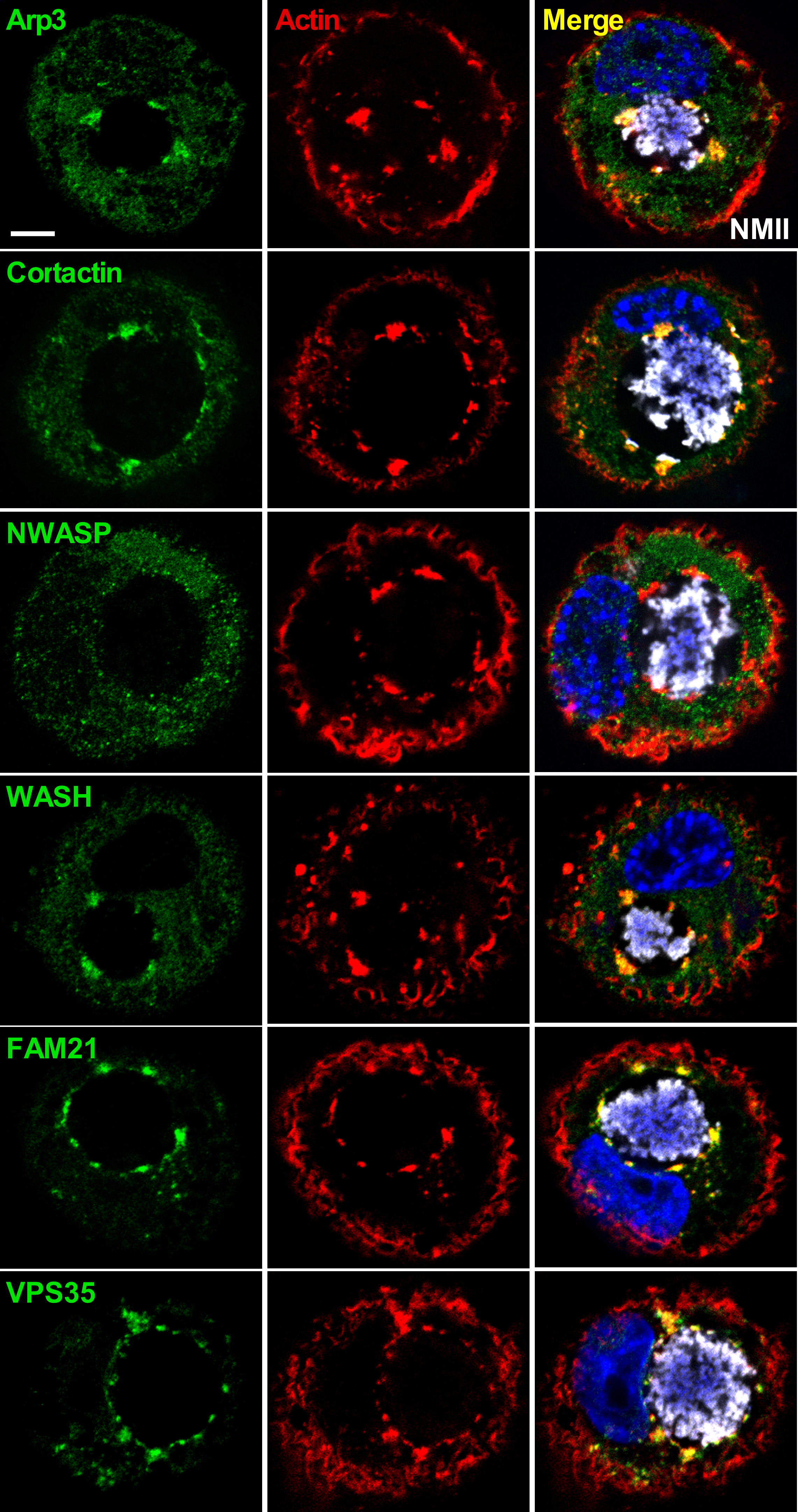

Supplement: S5 Fig — Three dpi THP-1 cells were immunostained for the indicated proteins. Colocalization of CCV actin patches is high for cortactin, Arp3, and the Arp2/3-promoting factors WASH and FAM21. The retromer protein VPS35 also colocalizes with patches. N-WASP shows no colocalization. NMII, C. burnetii Nine Mile phase II strain. Scale bar, 5 μm. (TIF) [file ppat.1007005.s005.tif]

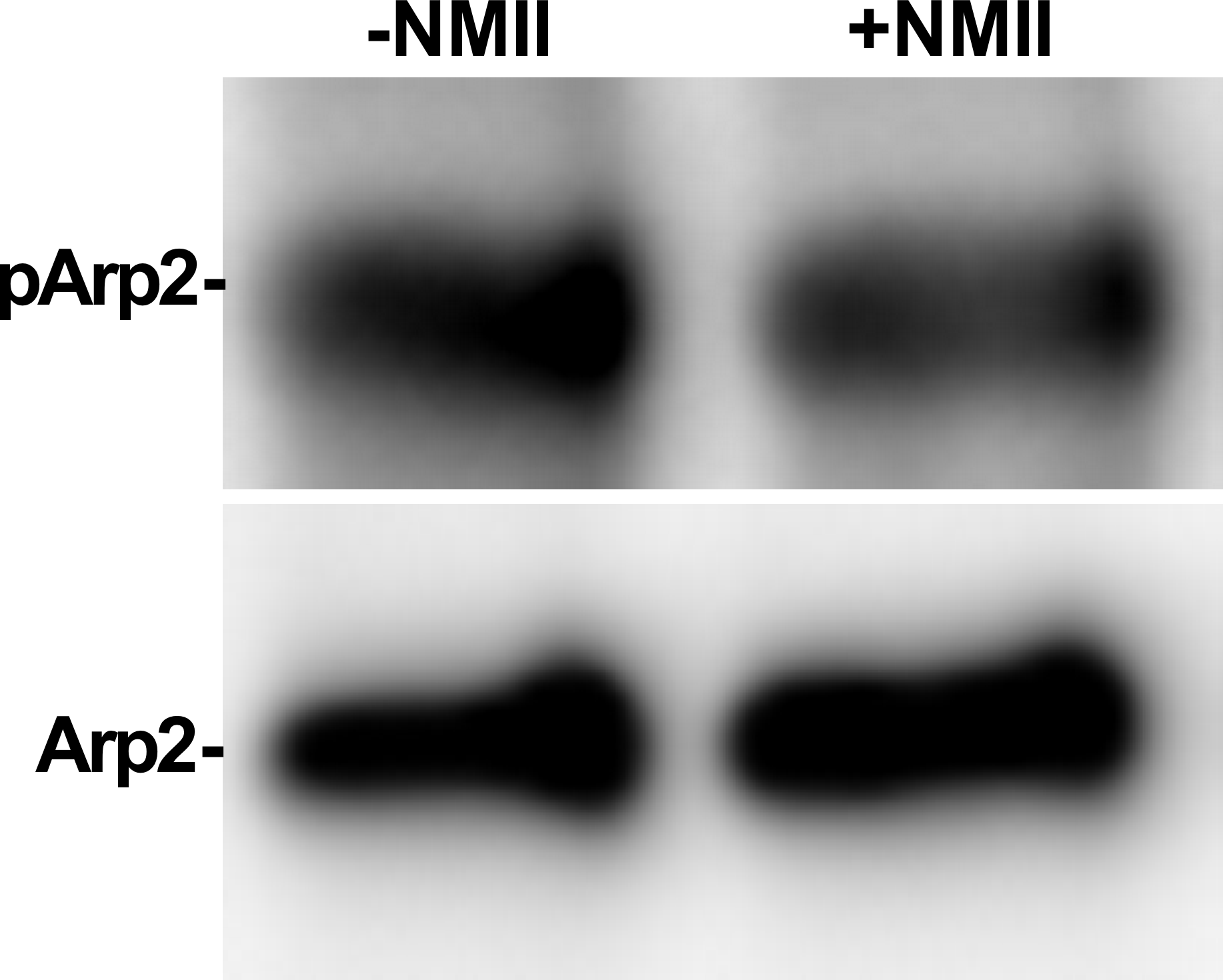

Supplement: S6 Fig — Immunoblot of lysates of infected or uninfected Vero cells grown for 3 days. The blot was probed with anti-phosphoArp2 antibody, then striped and probed anti-Arp2 antibody. (TIF) [file ppat.1007005.s006.tif]

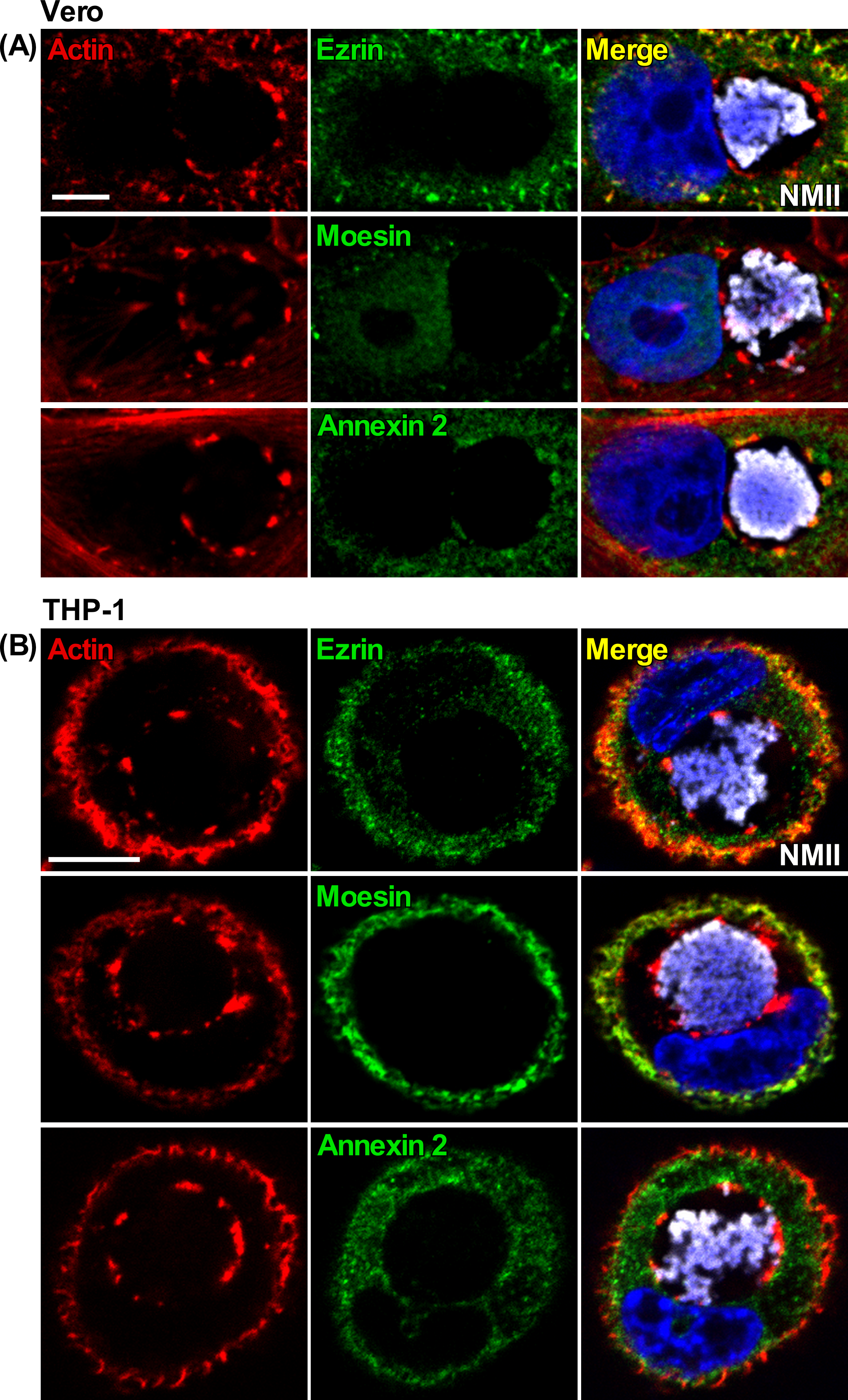

Supplement: S7 Fig — (A) Vero cells 3 dpi were immunostained for ezrin, moesin, or annexin 2. Ezrin and moesin have no colocalization with CCV actin patches whereas annexin 2 has poor colocalization that was not above background staining. (B) Same as (A), but with THP-1 cells. Neither ezrin, moesin, nor annexin 2 colocalize with CCV actin patches. NMII, C. burnetii Nine Mile phase II strain. Scale bar, 5 μm. (TIF) [file ppat.1007005.s007.tif]

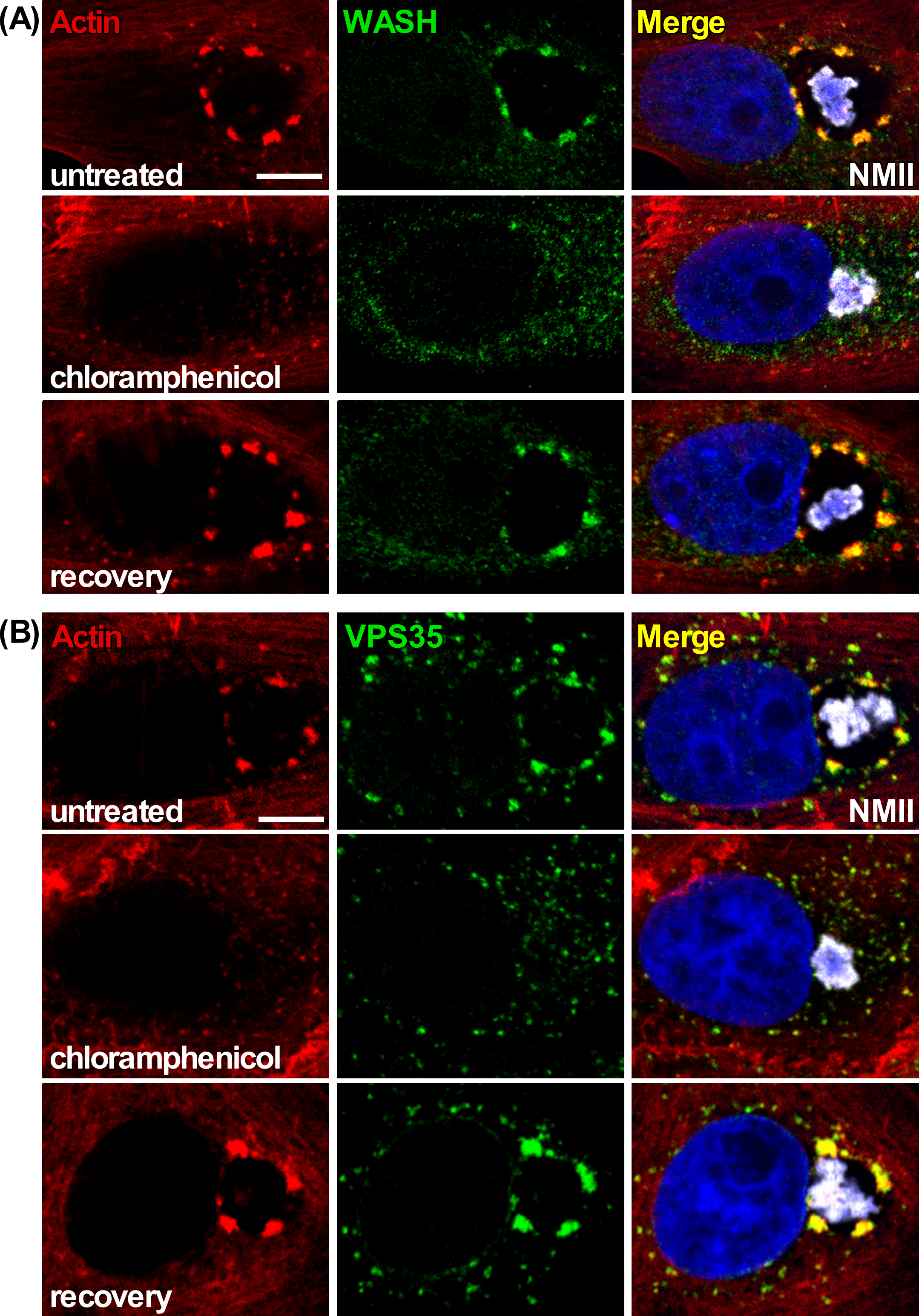

Supplement: S8 Fig — (A) Two dpi Vero cells treated with 50 μg/ml chloramphenicol for 24 hr were fixed along with the corresponding 3 dpi untreated controls, or washed to remove chloramphenicol and allowed an additional 24 hr recovery before fixation. Cells were stained for WASH and F-actin. Chloramphenicol treatment eliminates actin patches and WASH clusters on the CCV membrane (middle panel). (B) Same as (A) but stained for retromer component VPS35. VPS35 clustering on the CCV is also lost with chloramphenicol treatment. NMII, C. burnetii Nine Mile phase II strain. Scale bar, 5 μm. (TIF) [file ppat.1007005.s008.tif]

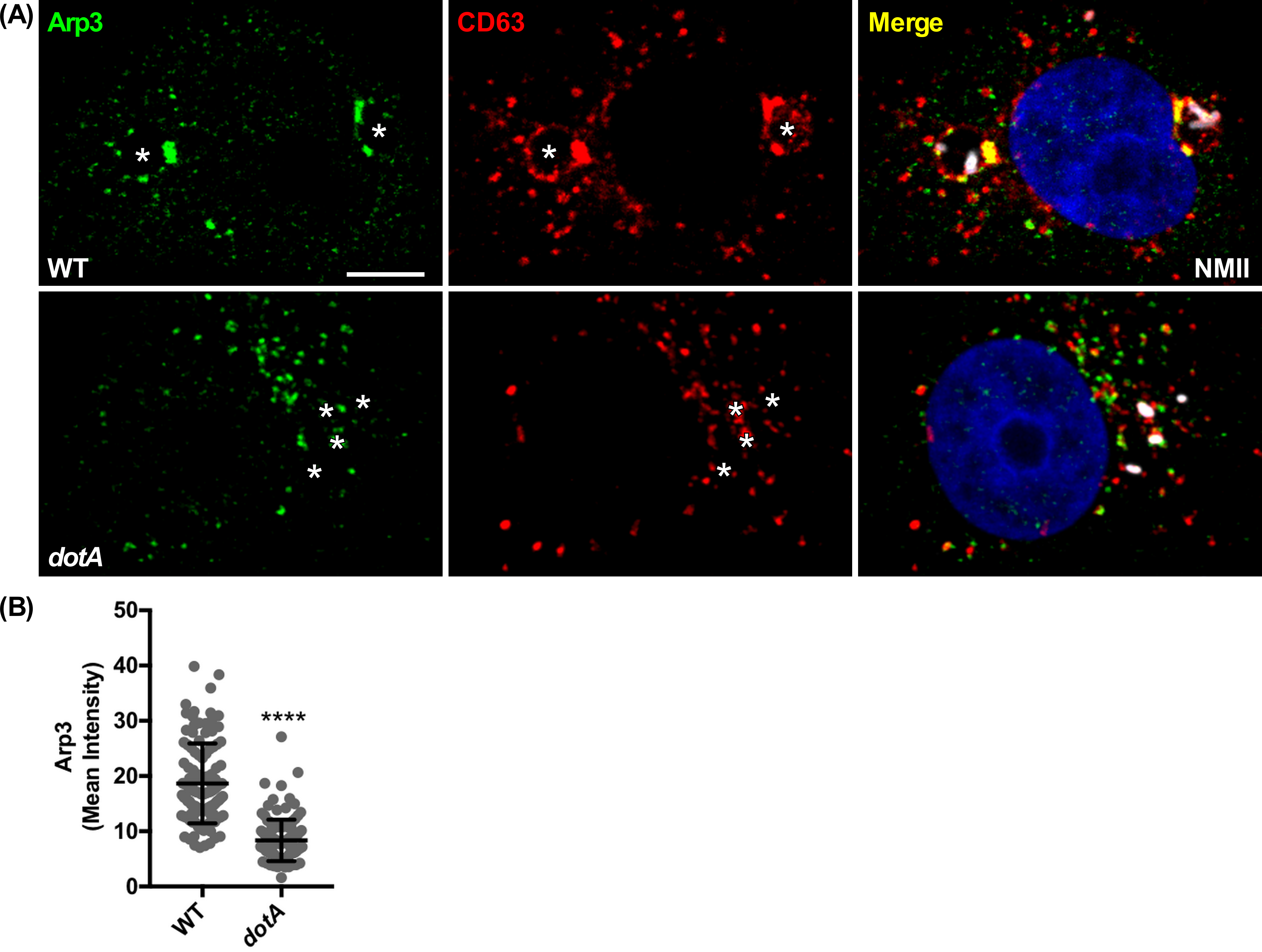

Supplement: S9 Fig — Vero cells infected for 24 hr with wild type C. burnetii or a dotA mutant were fixed and stained for CD63 and Arp3. Asterisks mark CCVs in Arp3 and CD63 panels. Histogram depicts the mean intensity of CCV Arp3 ± SD of ≥ 60 cells for at least 3 independent experiments. Statistical significance was determined using Student’s t-test (****P <0.0001). NMII, C. burnetii Nine Mile phase II strain. Scale bar, 5 μm. (TIF) [file ppat.1007005.s009.tif]

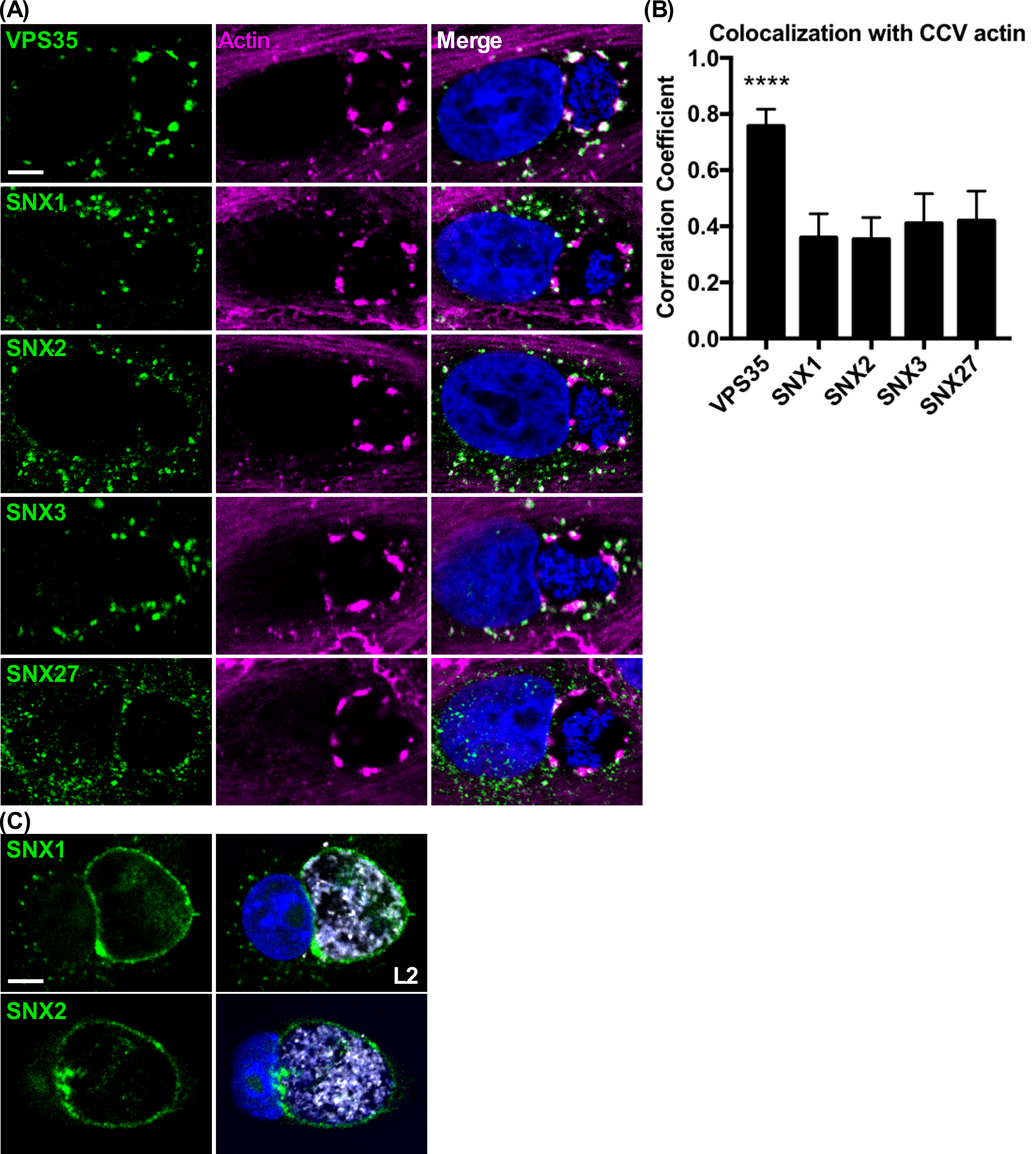

Supplement: S10 Fig — (A and B) Three dpi Vero cells were fixed, fluorescently stained for F-actin, and immunostained for the indicated sorting nexins (SNXs). SNXs show partial colocalization with CCV actin patches. (C) For a positive SNX control, Vero cells 1 dpi with Chlamydia trachomatis L2 were stained for SNX1 or SNX2. Similar to a previous report [48], SNX1 and SNX2 stain the C. trachomatis inclusion membrane, validating the antibodies. Colocalization analysis of CCVs was determined using Pearson’s correlation coefficient. Graphs represent the means ± SD of ≥ 60 cells from at least 3 independent experiments. Statistical significance was determined by the Student’s t-test (****P <0.0001). NMII, C. burnetii Nine Mile phase II strain. Scale bar, 5 μm. (TIF) [file ppat.1007005.s010.tif]

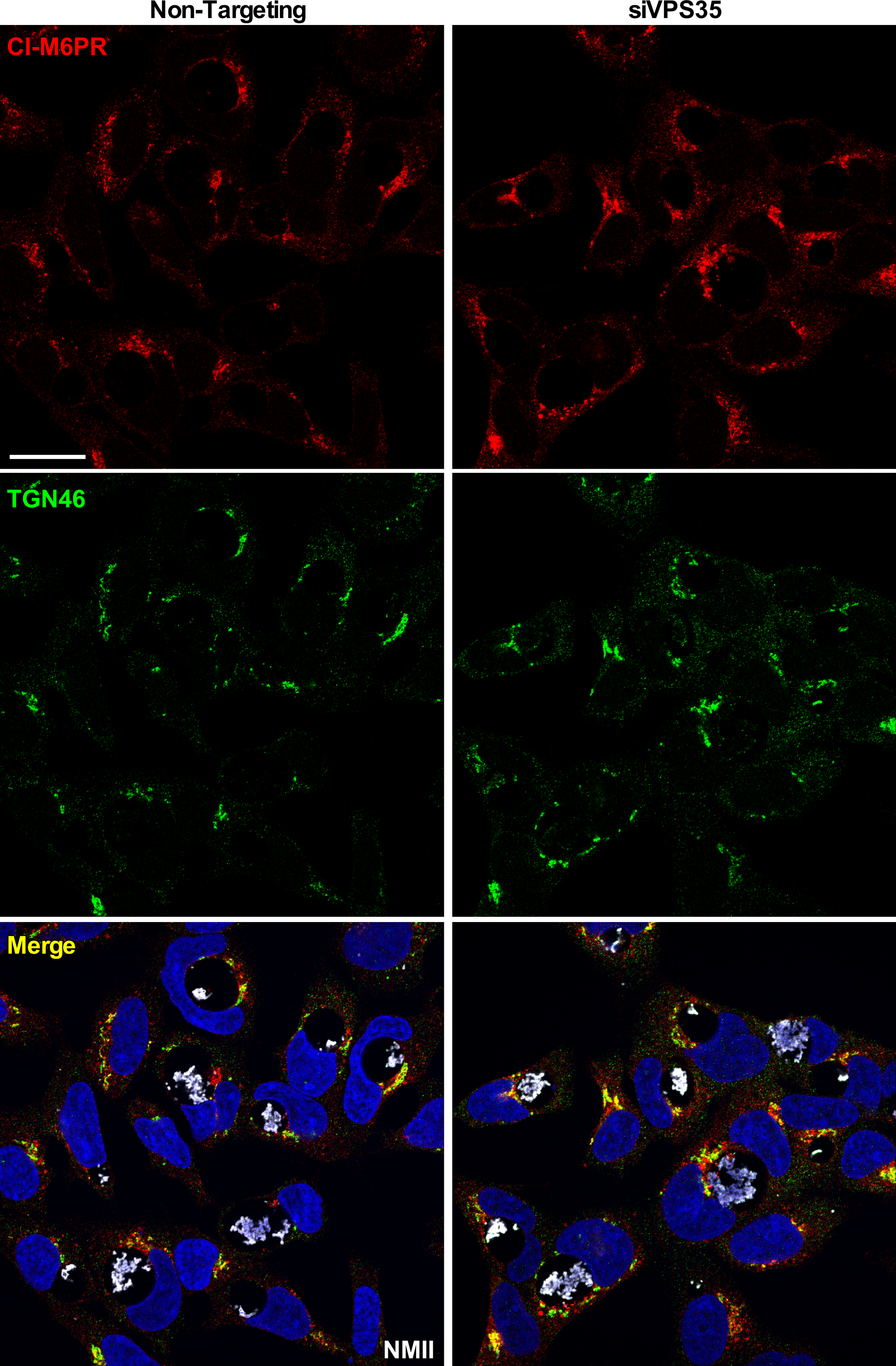

Supplement: S11 Fig — HEK 293 cells knocked down for VPS35 or non-targets and infected for 3 days were incubated with anti-CI-M6PR antibody for 1 hr at 37°C, then fixed and immunostained. The trans-Golgi (TNG46) was stained to evaluate retrograde trafficking of CI-M6PR. Knockdown of VPS35 (siVPS35) has no effect on CI-M6PR staining which shows focused colocalization with TGN46 similar to the non-targeted control. Images are representative of 2 independent experiments. NMII, C. burnetii Nine Mile phase II strain. Scale bar, 10 μm. (TIF) [file ppat.1007005.s011.tif]

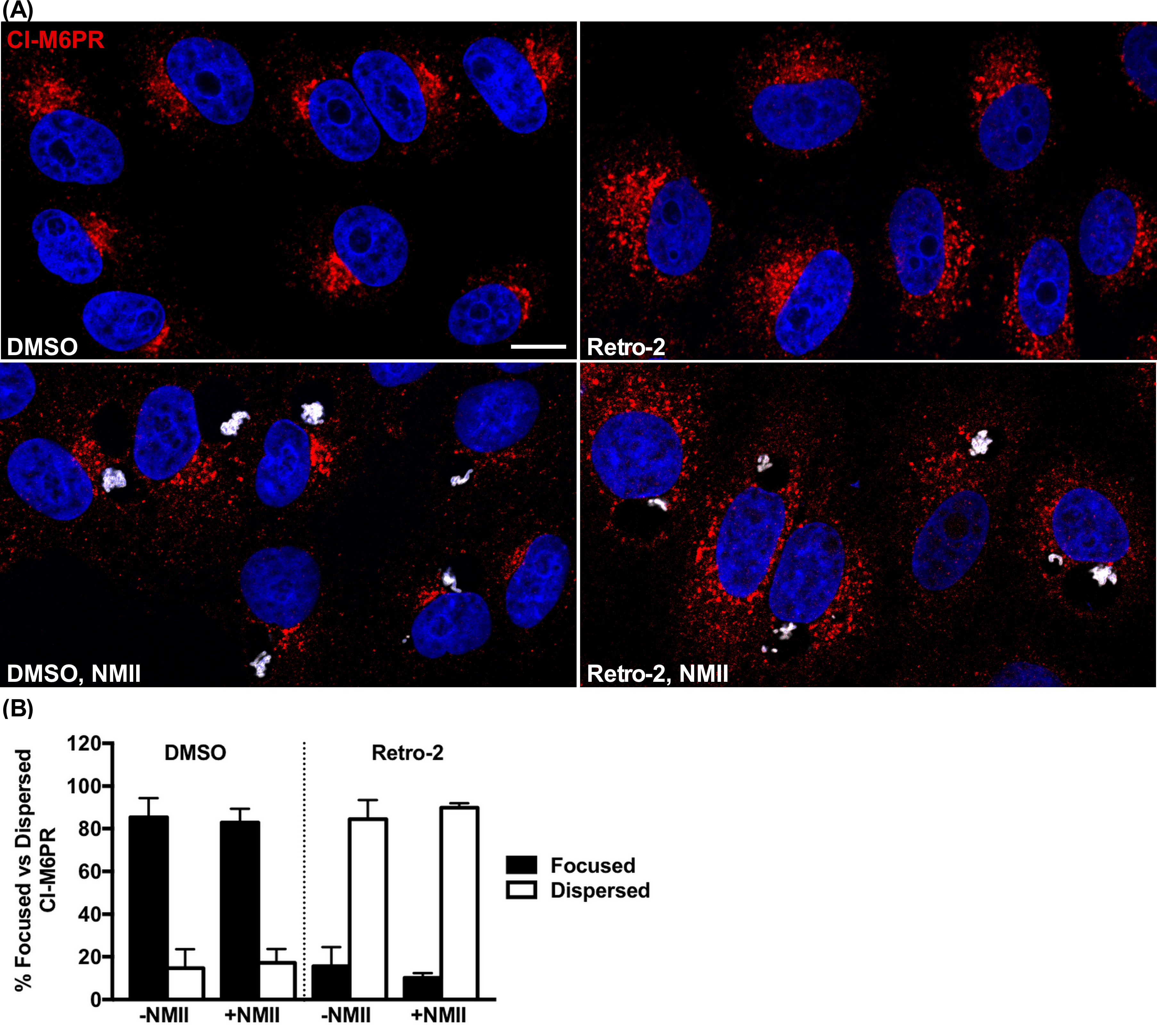

Supplement: S12 Fig — (A) Uninfected or 3 dpi Vero cells treated with DMSO or Retro-2 for 2 days (added 1 dpi) were incubated with anti-CI-M6PR antibody for 1 hr, then fixed and immunostained. (B) Images in (A) were analyzed for focused and dispersed CI-M6PR staining. In contrast to highly dispersed staining of CI-M6PR in Retro-2 treated cells, untreated infected and uninfected cells display focused peri-nuclear staining. Graphs represent the means ± SD of ≥ 50 cells from 3 independent experiments. NMII, C. burnetii Nine Mile phase II strain. Scale bar, 10 μm. (TIF) [file ppat.1007005.s012.tif]

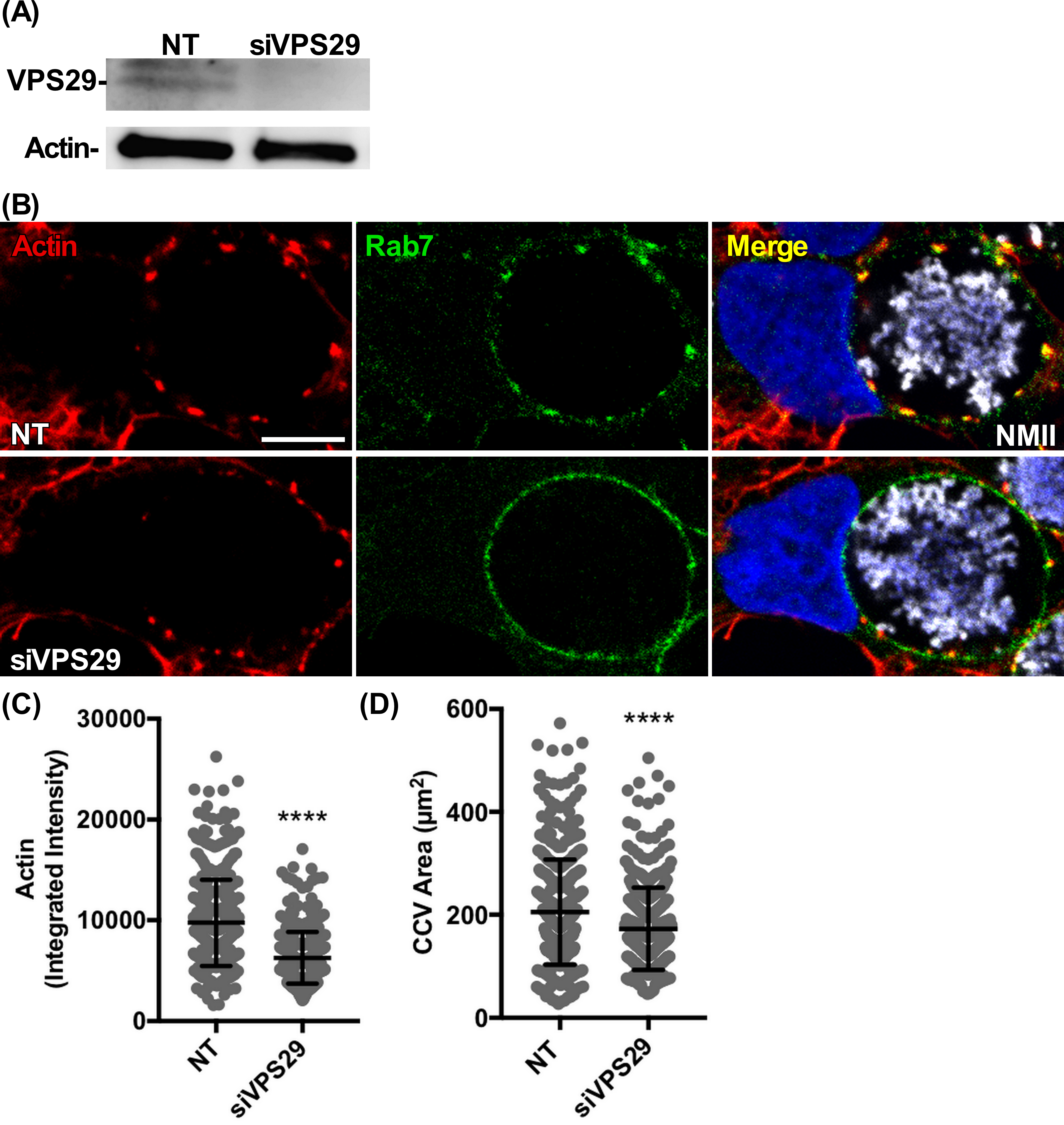

Supplement: S13 Fig — (A) Immunoblot of HEK 293 cells transfected with siRNA for VPS29 (siVPS29) or a non-targeting pool (NT). Actin was used as a loading control. (B-D) Three dpi VPS29 knockdown or NT treated HEK 293 cells were fluorescently stained for F-actin and Rab7. Knockdown of VPS29 redistributes Rab7 on the CCV and CCV actin patch size and intensity are decreased along with a slight reduction in CCV area. Graphs represent the means ± SD of ≥ 60 cells from 3 independent experiments. Statistical significance was determined by the Student’s t-test (****P <0.0001). NMII, C. burnetii Nine Mile phase II strain. Scale bar, 5 μm. (TIF) [file ppat.1007005.s013.tif]

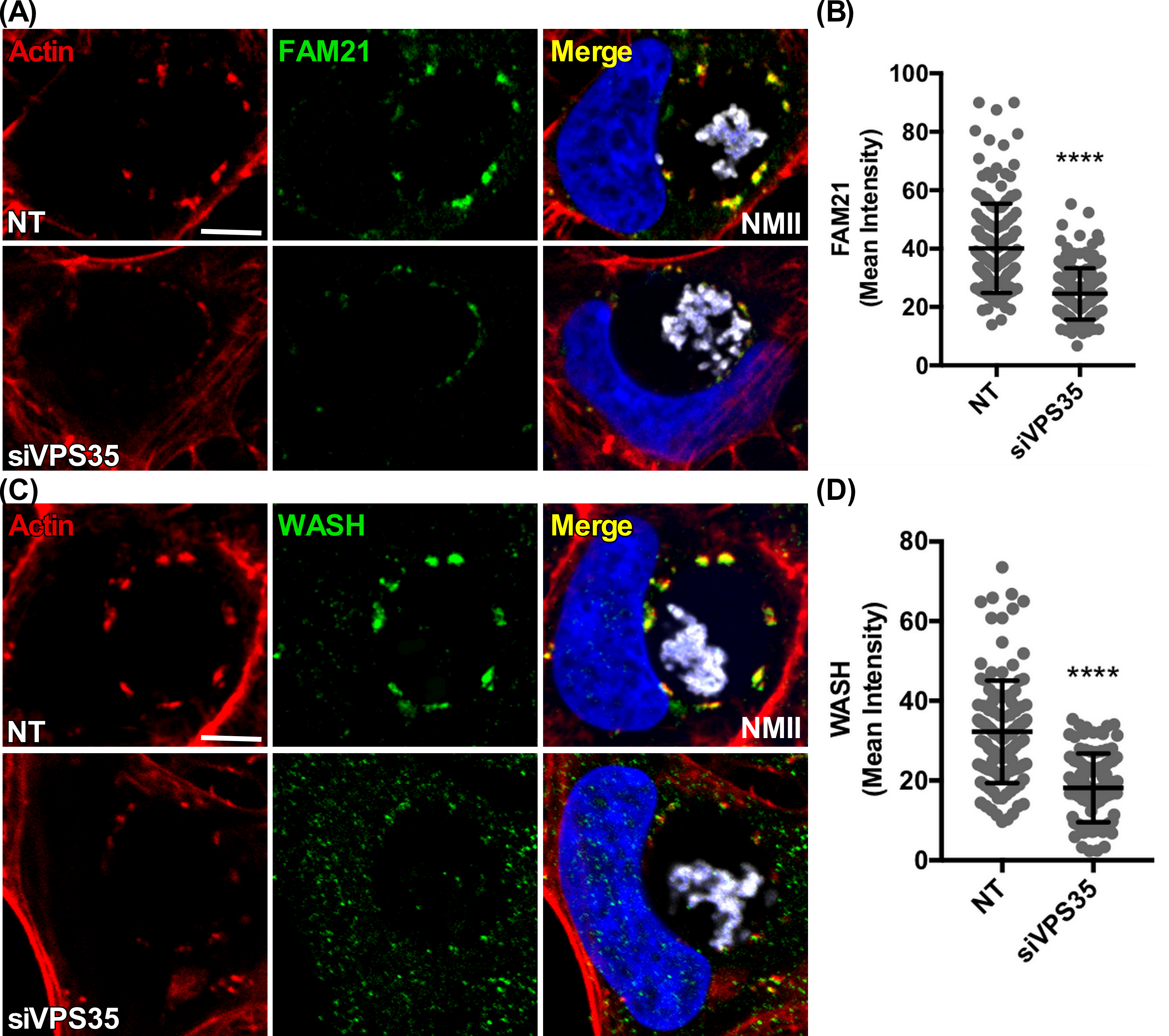

Supplement: S14 Fig — (A and B) Three dpi siVPS35 or NT treated HEK 293 cells were fixed and fluorescently stained for F-actin or FAM21. VPS35 knockdown cells have decreased FAM21 intensity on the CCV membrane. (C and D) Same as (A and B) but stained for WASH. Knockdown of VPS35 also reduces WASH intensity on the CCV membrane. Graphs represent the means ± SD of ≥ 60 cells from 3 independent experiments. Statistical significance determined by the Student’s t-test (****P <0.0001). NMII, C. burnetii Nine Mile phase II strain. Scale bar, 5 μm. (TIF) [file ppat.1007005.s014.tif]

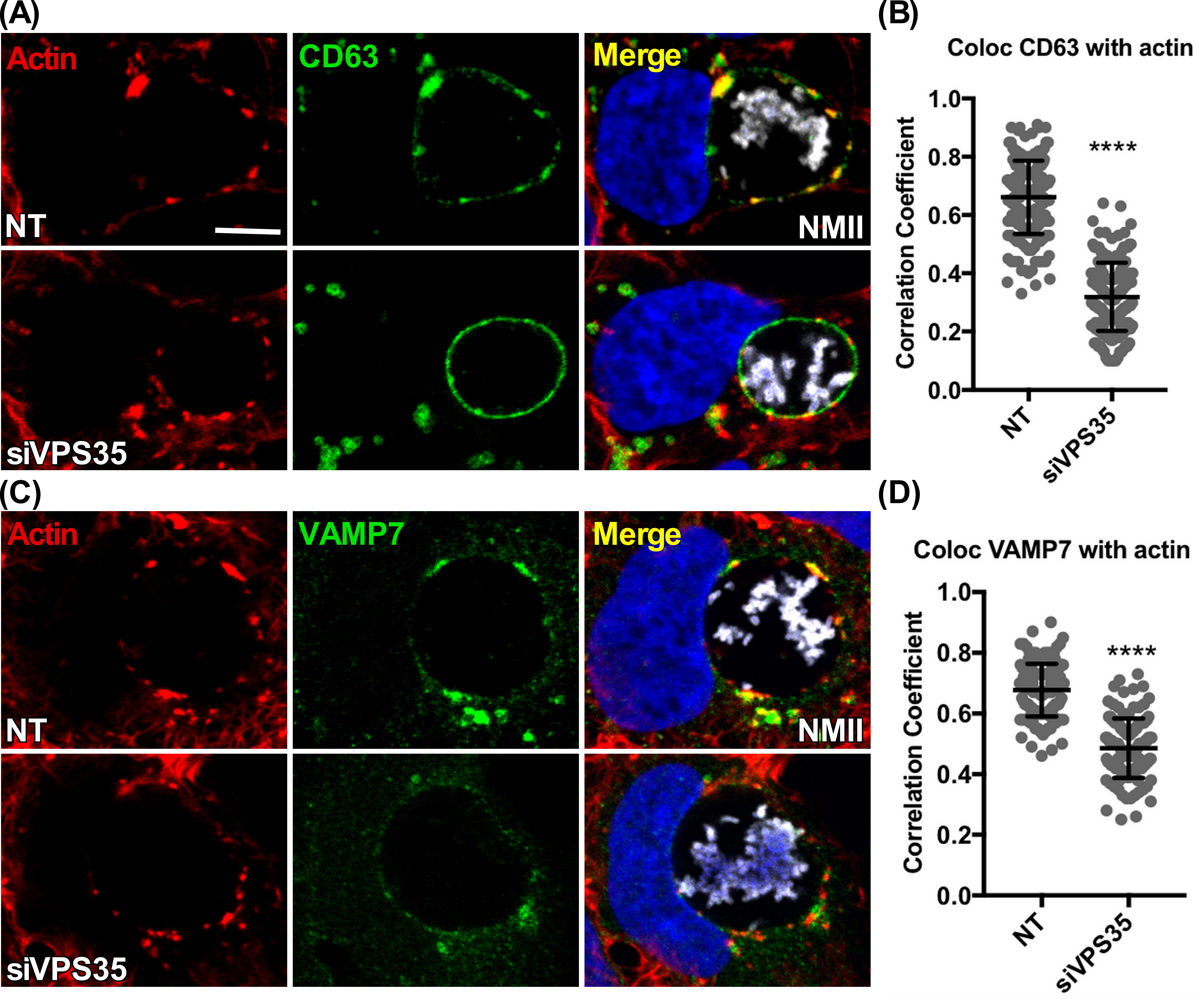

Supplement: S15 Fig — (A and B) VPS35 knockdown or control HEK 293 cells at 3 dpi were fluorescently stained for F-actin and CD63. VPS35 knockdown cells have uniform staining of CD63 around the CCV and lack colocalization of CD63 clusters with reduced CCV actin patches. (C and D) Same as (A and B) but with VAMP7 staining. VAMP7 staining is still observed around CCVs of VPS35 knockdown cells but lacks colocalization with reduced CCV actin patches. Graphs represent the means ± SD of ≥ 60 cells from 3 independent experiments. Colocalization was determined using Pearson’s correlation coefficient. Statistical significance was determined by the Student’s t-test (****P <0.0001). NMII, C. burnetii Nine Mile phase II strain. Scale bar, 5 μm. (TIF) [file ppat.1007005.s015.tif]

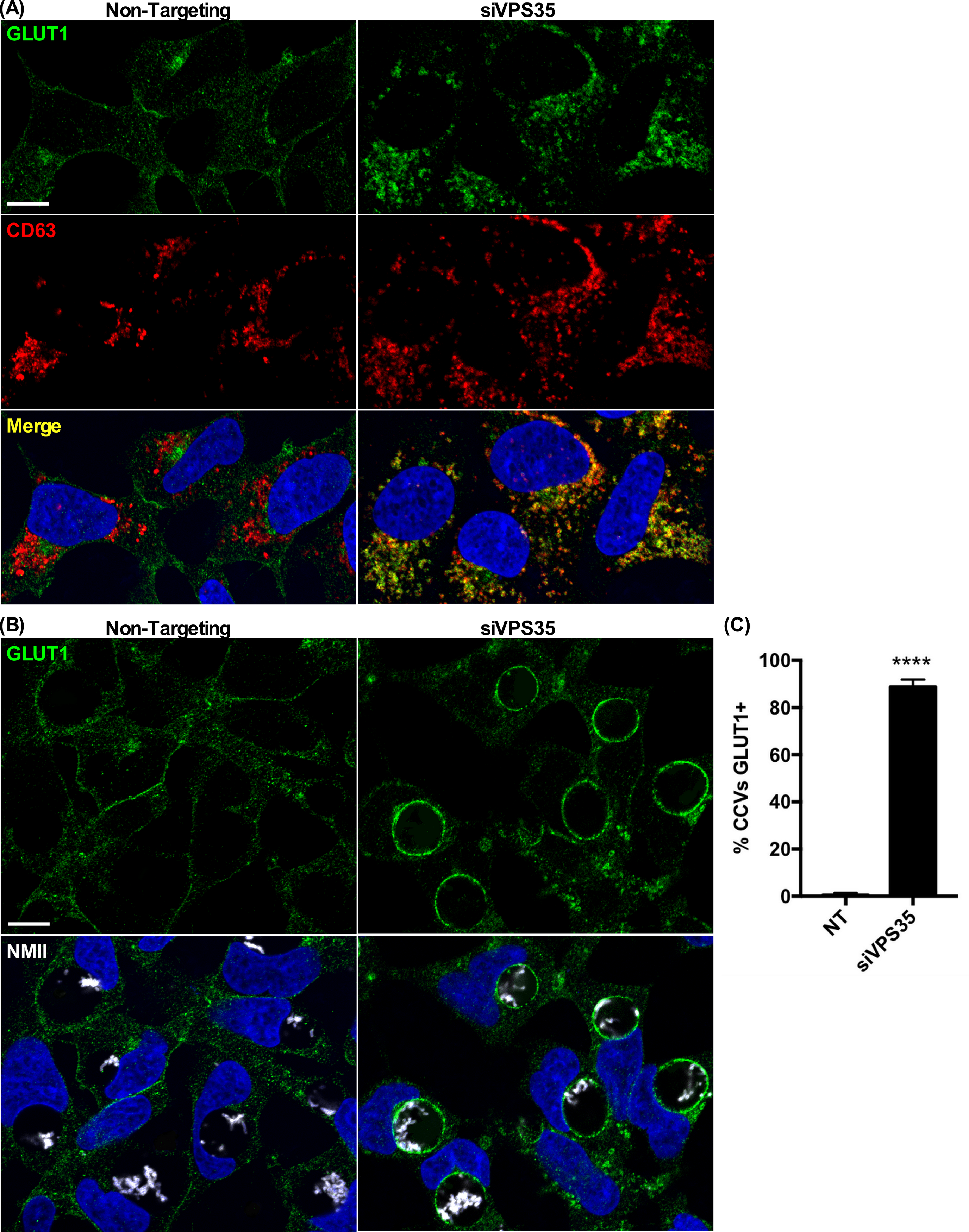

Supplement: S16 Fig — (A) Uninfected VPS35 knockdown or NT-treated HEK 293 cells were stained for GLUT1 and CD63. As previously reported [57], knockdown of VPS35 disrupts recycling from early endosomes to the plasma membrane, redirecting GLUT1 trafficking to late endosomes/lysosomes. (B and C) Three dpi VPS35 knockdown or NT-treated HEK 293 cells were fixed and stained for GLUT1. Infection alone does not affect GLUT1 trafficking. However, knockdown of VPS35 results in trafficking of GLUT1+ vesicles to the CCV, increasing GLUT1 intensity on the CCV membrane. Graphs represent the means ± SD of ≥ 60 cells from 3 independent experiments. Statistical significance was determined by the Student’s t-test (****P <0.0001). NMII, C. burnetii Nine Mile phase II strain. Scale bar, 5 μm. (TIF) [file ppat.1007005.s016.tif]

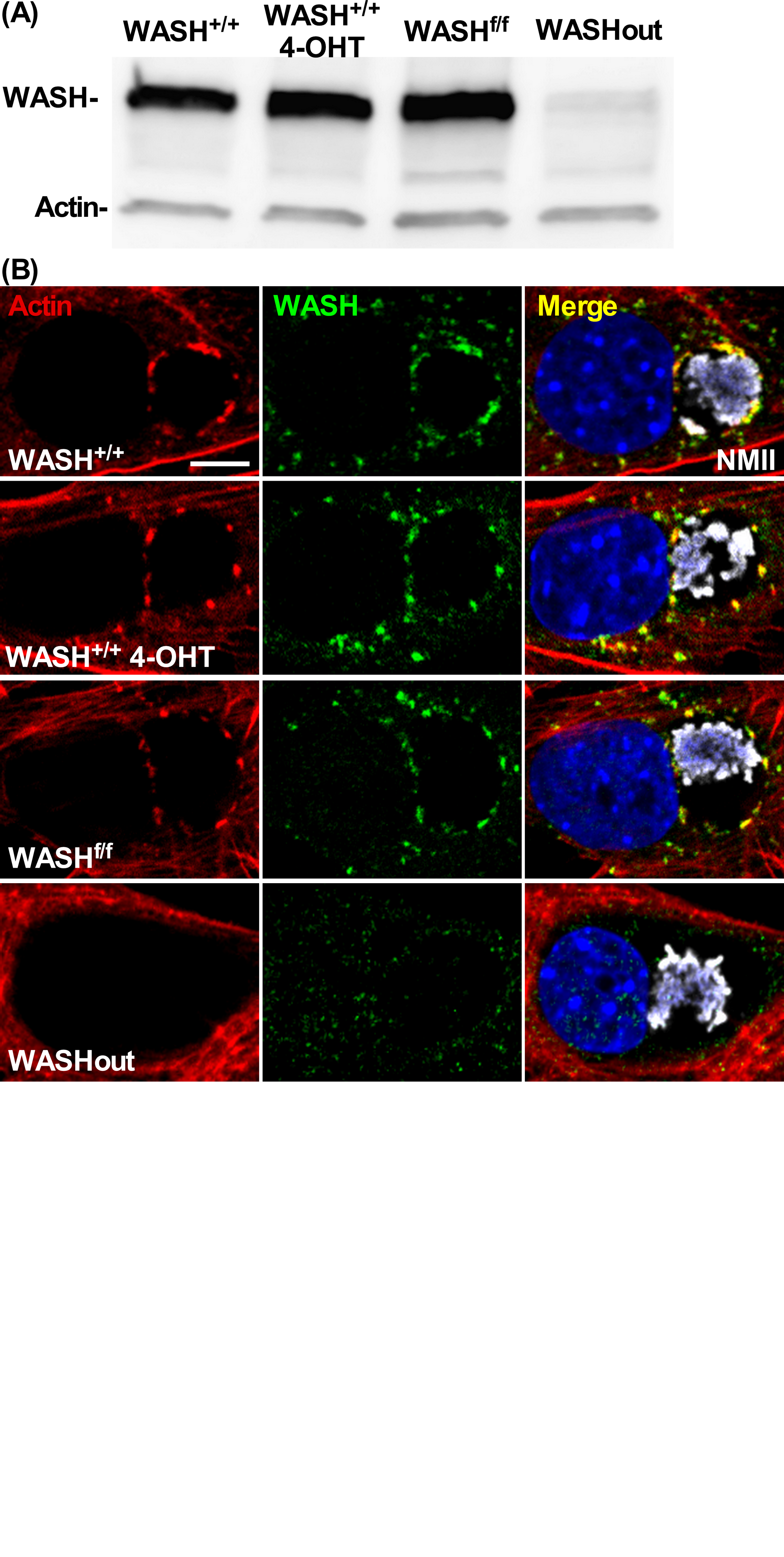

Supplement: S17 Fig — (A) Immunoblot of lysates of control wild type MEFs (WASH+/+), untreated or treated with 4-hydroxy-tamoxifen (4-OHT), and WASHf/f MEFs, untreated or treated with 4-OHT (WASHout). Actin served as a loading control. (B) Three dpi MEFs fluorescently stained for F-actin and WASH. WASHout MEFs do not have CCV actin patches, but still support C. burnetii growth. NMII, C. burnetii Nine Mile phase II strain. Scale bar, 5 μm. (TIF) [file ppat.1007005.s017.tif]

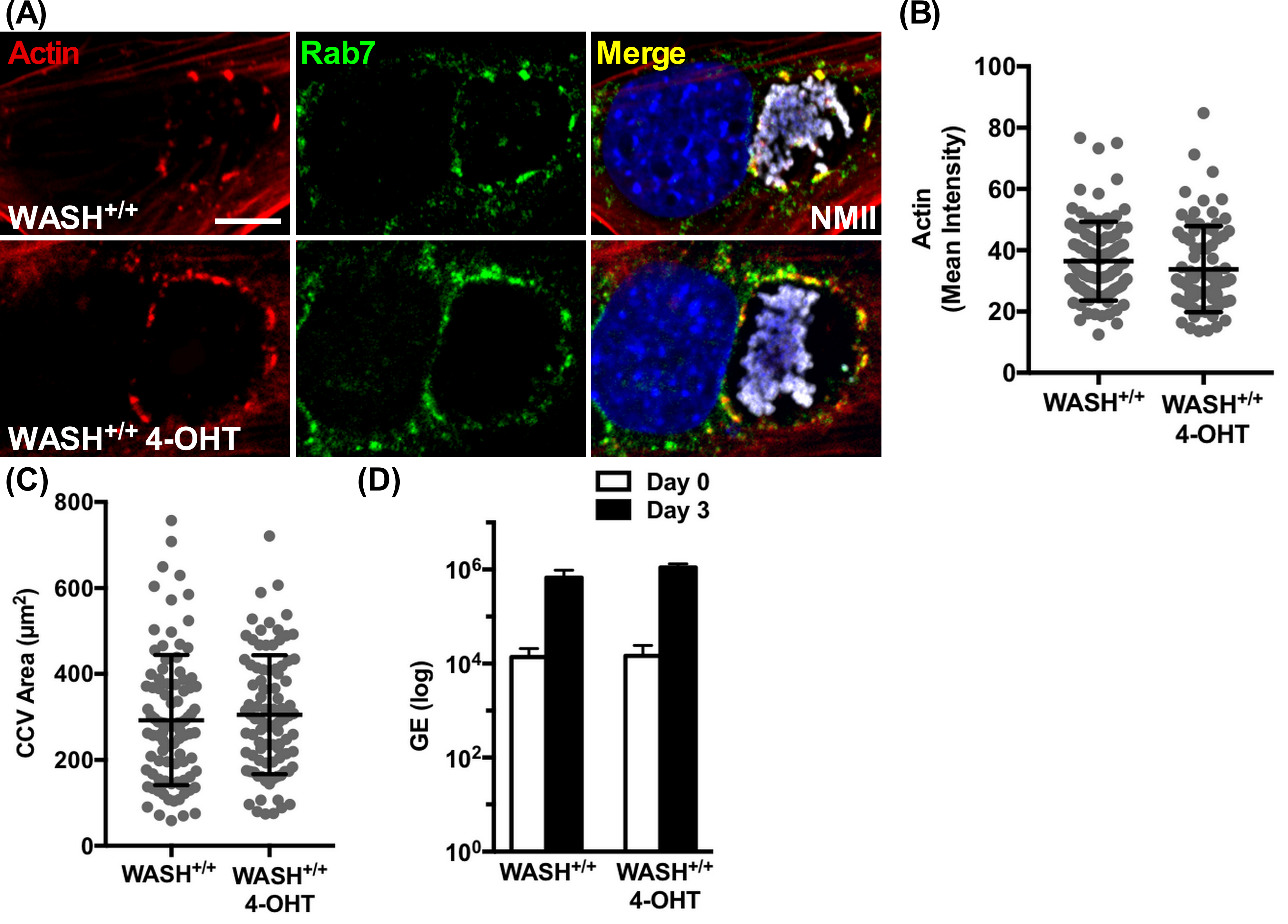

Supplement: S18 Fig — (A) Wild type WASH+/+ MEFs untreated or treated with 4-OHT were fixed 3 dpi and fluorescently stained for F-actin and Rab7. Treatment of 4-OHT had no effect on CCV actin patch formation or Rab7 clustering on the CCV. (B and C) F-actin intensity and areas of CCVs from images of (A). Untreated and 4-OHT treated MEFs have similar CCV actin patch intensity and CCV areas. (D) Growth analysis of C. burnetii in WASH+/+ MEFs untreated or treated with 4-OHT using qPCR for determining genome equivalents (GE). No difference in C. burnetii replication is observed with 4-OHT treated cells. Graphs represent the means ± SD of ≥ 50 cells from 3 independent experiments. NMII, C. burnetii Nine Mile phase II strain. Scale bar, 5 μm. (TIF) [file ppat.1007005.s018.tif]

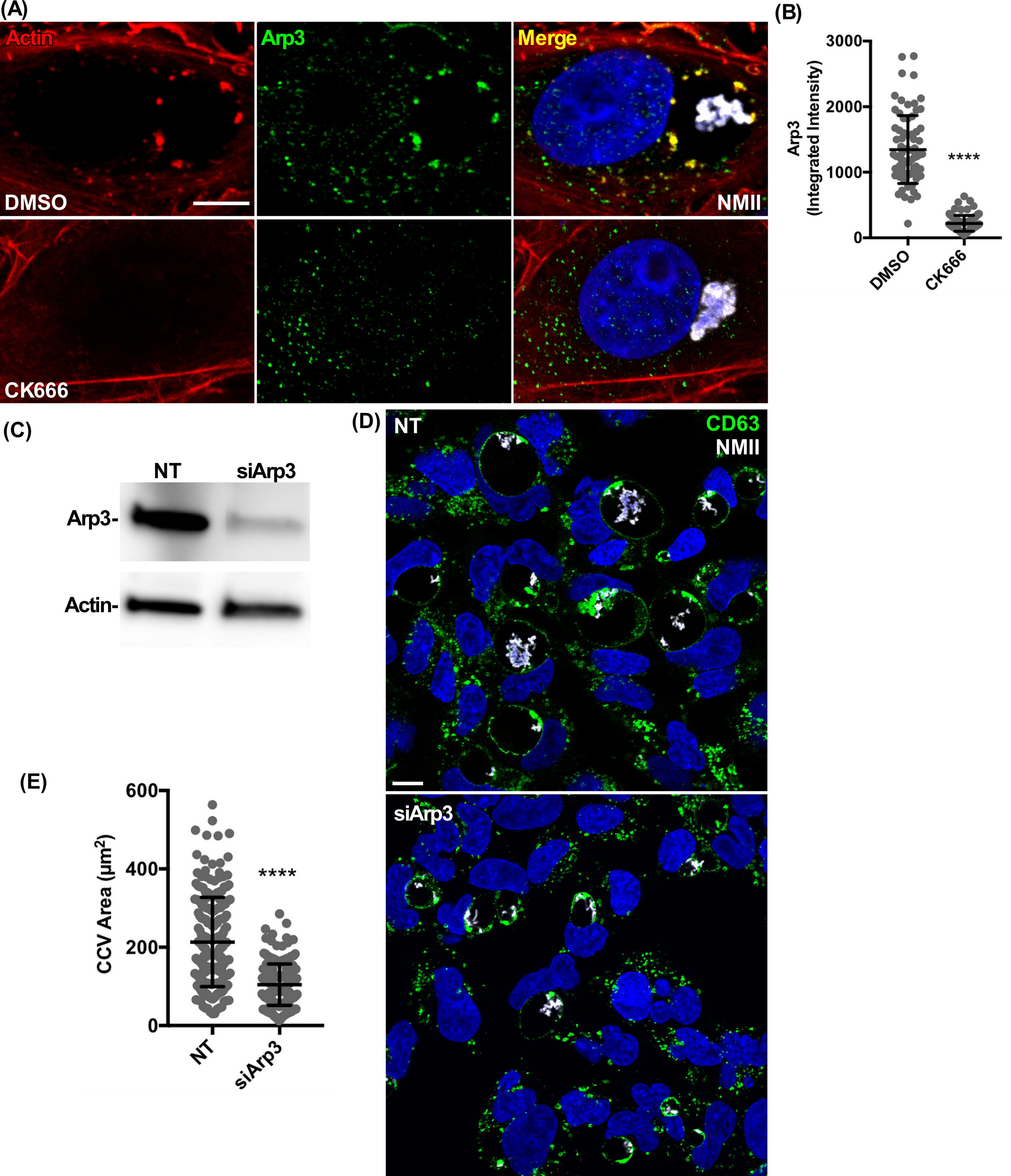

Supplement: S19 Fig — (A) Vero cells at 2 dpi were treated with CK-666 for 1 day. Cells were fixed and fluorescently stained for actin and Arp3. (B) Histogram depicts the integrated intensity of CCV Arp3 ± SD of ≥ 60 cells for at least 3 independent experiments. Statistical significance was determined using Student’s t-test (****P <0.0001). NMII, C. burnetii Nine Mile phase II strain. Scale bar, 5 μm. (C) Immunoblot of HEK 293 cells with knockdown of Arp3 by siRNA. A non-targeting pool (NT) siRNA was included. Actin was used as a loading control. (D) Three dpi Arp3 knockdown or NT-treated HEK 293 cells fluorescently stained for CD63. (E) Histogram depicts the CCV area ± SD of ≥ 60 cells for at least 3 independent experiments. Statistical significance was determined by the Student’s t-test (****P <0.0001). NMII, C. burnetii Nine Mile phase II strain. Scale bar, 5 μm. (TIF) [file ppat.1007005.s019.tif]

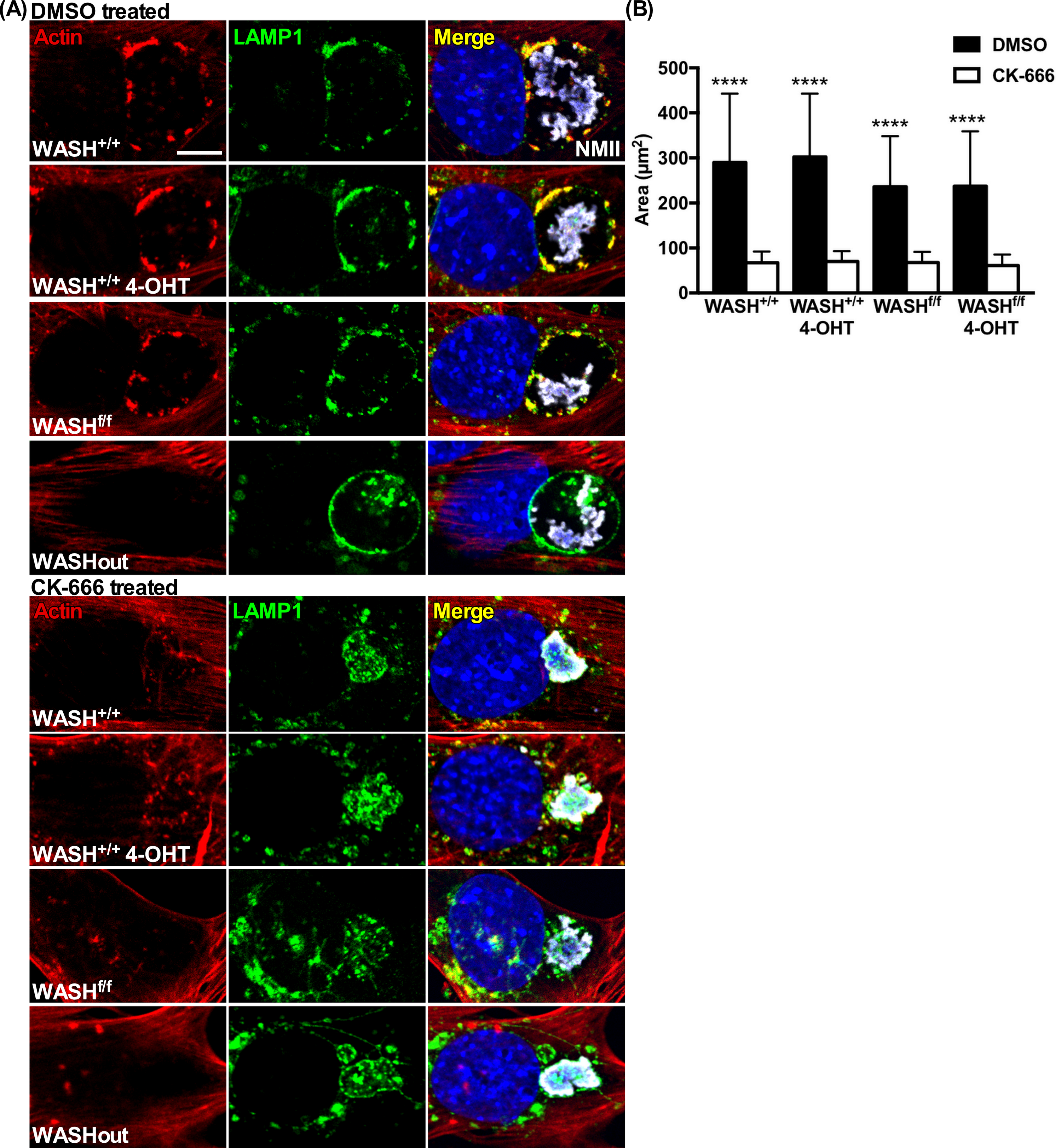

Supplement: S20 Fig — (A and B) WASH+/+ or WASHf/f MEFs untreated or 4-OHT treated were incubated with DMSO or CK-666 2 dpi for 24 hr, then fluorescently stained for LAMP1 and F-actin. The area of individual CCVs in CK-666 treated cells was measured and compared to DMSO-treated cells. CCVs in WASHout MEFs, which do not require actin patches for lysosomal fusion, have reduced CCV area with CK-666 treatment. Graph represents the means ± SD of ≥ 60 cells from 3 independent experiments. Statistical significance was determined by the Student’s t-test (****P <0.0001). NMII, C. burnetii Nine Mile phase II strain. Scale bar, 5 μm. (TIF) [file ppat.1007005.s020.tif]

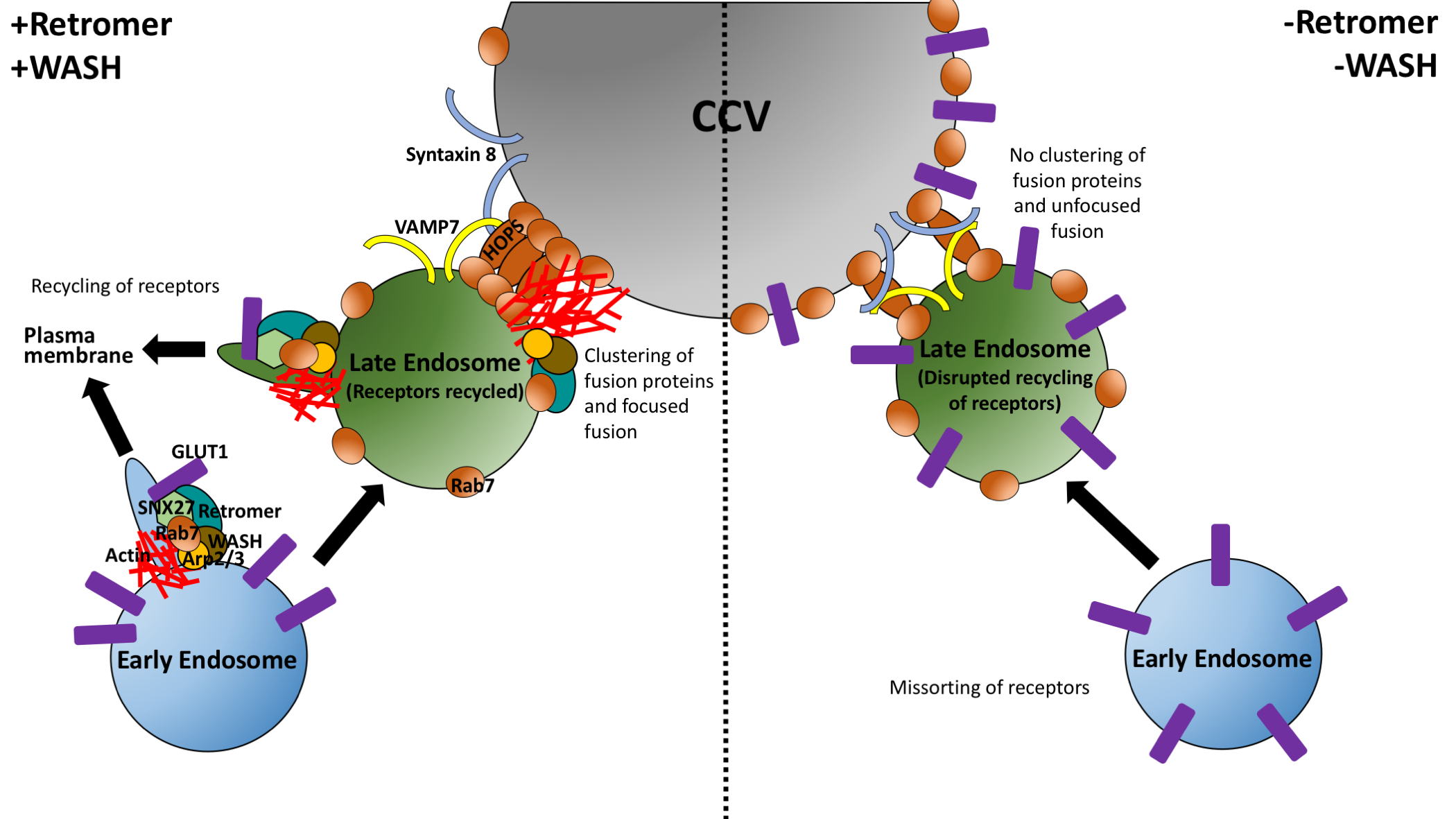

Supplement: S21 Fig — The proposed model depicts a dual regulatory role for Rab7 in mediating recruitment of retromer for membrane receptor recycling and fusion of late endosomes with the Coxiella-containing vacuole (CCV). In the presence of retromer and WASH (left of dashed line), GLUT1 on transitioning early to late endosomes is normally recycled to the plasma membrane. GLUT1 is recognized by SNX27, which along with Rab7, recruits the retromer-WASH-Arp2/3 complex that generates F-actin required for recycling. Simultaneously, Rab7 recruits the retromer-WASH-Arp2/3 complex which generates F-actin sorting platforms that cluster Rab7 on the CCV. In turn, HOPs/SNARE complexes (VAMP7 and syntaxin 8) are recruited to platforms which results in focused fusion of late endosomes with the CCV. In the absence of retromer and actin sorting platforms (right of dashed line), the unfocused nature of Rab7 results in GLUT1 receptors that are not recycled but instead deposit on the CCV. Late endosomal markers also disperse in the CCV due to the unfocused nature of Rab7. (TIF) [file ppat.1007005.s021.tif]
